# ElixirSeeker: A Machine Learning Framework Utilizing Fusion Molecular Fingerprints for the Discovery of Lifespan‐Extending Compounds

**DOI:** 10.1111/acel.70116

**Published:** 2025-05-26

**Authors:** Yan Pan, Hongxia Cai, Fang Ye, Wentao Xu, Zhihang Huang, Jingyuan Zhu, Yiwen Gong, Yutong Li, Anastasia Ngozi Ezemaduka, Shan Gao, Shunqi Liu, Guojun Li, Hao Li, Jing Yang, Junyu Ning, Bo Xian

**Affiliations:** ^1^ Department of Neurology, Sichuan Provincial People's Hospital, School of Medicine University of Electronic Science and Technology of China Chengdu China; ^2^ Laboratory of Aging Research, School of Medicine University of Electronic Science and Technology of China Chengdu China; ^3^ Department of Biochemistry and Biophysics University of California San Francisco California USA; ^4^ Institute for Toxicology, Beijing Center for Disease Prevention and Control Beijing China; ^5^ College of Animal Science and Technology, China Agricultural University Beijing China; ^6^ School of Public Health, Capital Medical University Beijing China

**Keywords:** aging, *Caenorhabditis elegans*, drug discovery, lifespan‐extending, machine learning

## Abstract

Despite the growing interest in developing anti‐aging drugs, high costs and low success rates of traditional drug discovery methods pose significant challenges. Aging is a complex biological process associated with numerous diseases, making the identification of compounds that can modulate aging mechanisms critically important. Accelerating the discovery of potential anti‐aging compounds is essential to overcome these barriers and enhance lifespan and healthspan. Here, we present ElixirSeeker, a machine learning framework designed to maximize feature capture of lifespan‐extending compounds through multi‐fingerprint fusion mechanisms. Utilizing this approach, we identified several promising candidate drugs from external compound databases. We tested the top six hits in *Caenorhabditis elegans* and found that four of these compounds—including Praeruptorin C, Polyphyllin VI, Thymoquinone, and Medrysone—extended the organism's lifespan. This study demonstrates that ElixirSeeker effectively accelerates the identification of viable anti‐aging compounds, potentially reducing costs and increasing the success rate of drug development in this field.

## Graphical abstract

ElixirSeeker is a machine learning architecture based on a phenotype‐driven drug discovery approach to screen lifespan‐extending compounds. By integrating molecular fingerprints, ElixirSeeker could maximize feature capture of lifespan‐extending compounds. We tested the top six hits in *Caenorhabditis elegans* and found that four of these compounds extended the organism's lifespan.

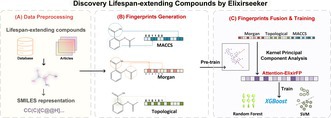

## 1 Introduction

**FIGURE 1:**
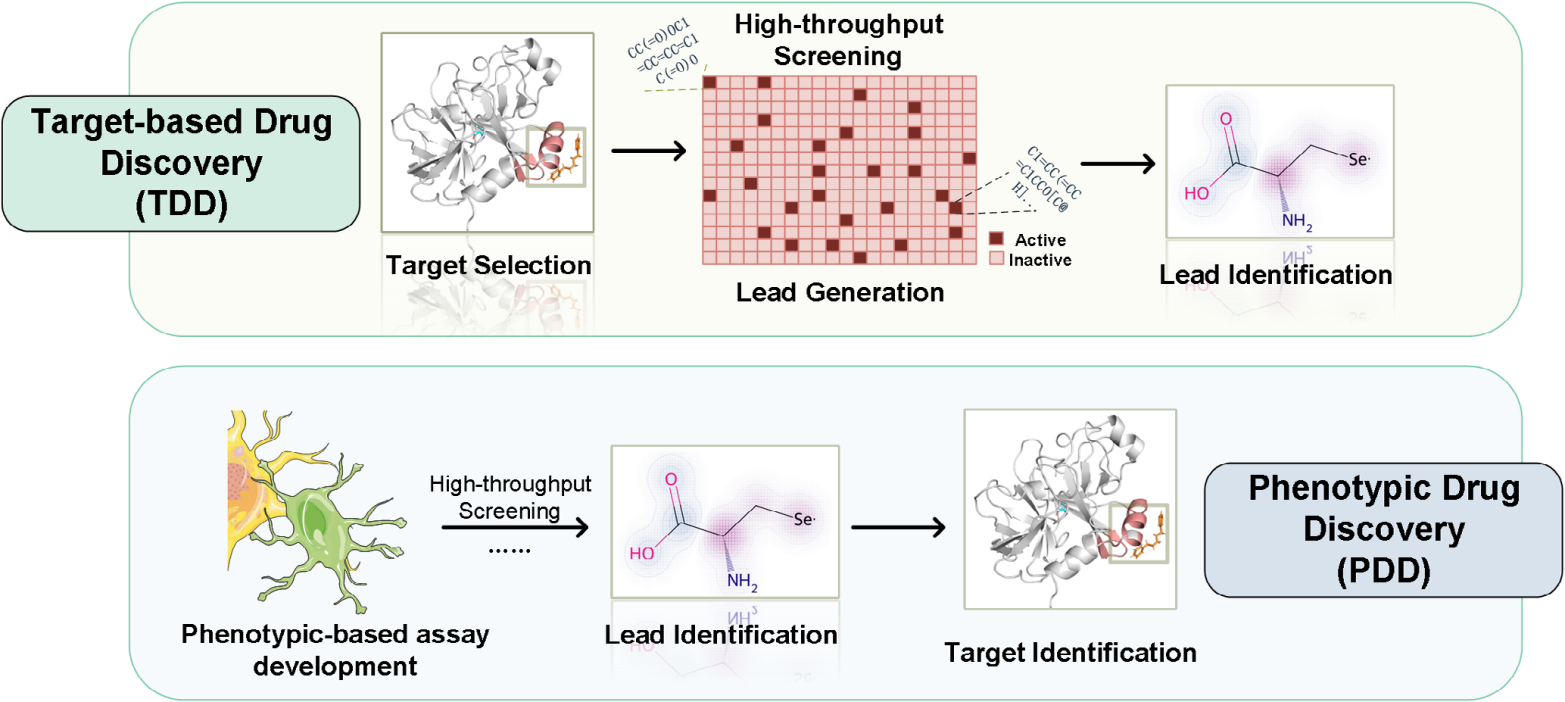
Overview of target‐based drug discovery (TDD) and phenotypic drug discovery (PDD).

The demand for anti‐aging products and preventive medicines is constantly rising as the worldwide population ages. Various pharmacological approaches have been shown to increase lifespan and attenuate age‐related diseases (Scheen 2018; Zhu et al. 2019; Dehghan et al. 2019; Strong et al. 2020; Cabreiro et al. 2013; Singh et al. 2023; Chaib et al. 2022; Martel, Ojcius, et al. 2020). As anti‐aging pharmacology advances, high cost and low success rate hinder drug development, emphasizing the need for identifying more promising candidate compounds. In the past, the focus has been on target‐based drug discovery (TDD), which designs compounds for specific targets. The demand for more novel and promising anti‐aging, especially lifespan‐extending, drug candidates would require phenotypic drug discovery (PDD), a target‐agnostic approach focusing on the phenotypic impacts of treatments in relevant biological systems (Figure 1).

As a classical model organism, the nematode, *Caenorhabditis elegans* ( *C. elegans* ), is extensively utilized in anti‐aging research. This organism is particularly valuable due to its short lifespan, simple genome, ease of manipulation, and significant genomic similarities to humans (Shen et al. 2018). Several studies have utilized *C. elegans* to identify numerous small molecules that potentially extend lifespan and improve health. This model has facilitated the initial screening of hundreds of lifespan‐extending compounds (Banse et al. 2024). For example, research has demonstrated that natural products such as plant‐based substances (Martel, Wu, et al. 2020; Kim and Lee 2019), polyphenols (Liu et al. 2021), and herbal mixtures (Moriwaki et al. 2013) have significant potential for extending the lifespan extension of *C. elegans* .

Nevertheless, translating these findings into clinical candidates remains hampered by intrinsic challenges: Aging‐related datasets are characteristically sparse, noisy, and contaminated by false positives—a reflection of biological complexity and methodological variability across studies. These data limitations render conventional deep learning approaches suboptimal, as they typically require large‐scale annotated datasets to achieve robust generalization.

Computational chemistry has revolutionized early drug discovery through machine learning‐driven bioactivity prediction (Jayatunga et al. 2022; Yuan et al. 2023), target deconvolution (Abbasi Mesrabadi et al. 2023; Amiri Souri et al. 2022; Ahn et al. 2022), and virtual screening (da Silva Rocha et al. 2019). Particularly, molecular fingerprint‐based algorithms have shown promise in metabolomic profiling (Issa et al. 2021; Ehiro 2024; de Fernánz‐ Gortari et al. 2017). However, critical limitations persist: (1) Arbitrary fingerprint length selection risks information loss or noise amplification (Yin et al. 2019; Sherwani et al. 2024; Zahid et al. 2024; Liu et al. 2024); (2) Current methodologies treat all molecular substructures as equally informative, neglecting context‐dependent biological relevance; and (3) Static fingerprint representations fail to adapt to specific therapeutic domains like geroscience.

To address these challenges, we present ElixirSeeker—a new machine learning framework integrating importance‐guided fingerprint fusion with ensemble learning. Our methodology strategically combines three elements: First, an XGBoost‐based meta‐learner dynamically determines optimal fingerprint lengths while assigning attention weights to critical substructures. Second, Kernel PCA (KPCA) enables nonlinear dimensionality reduction tailored to lifespan‐extending compound spaces. Third, iterative feature importance scoring creates fused fingerprint representations that synergistically capture complementary chemical information. This ensemble approach proves particularly advantageous for aging‐related datasets where limited positive samples (*n* = 276 curated compounds in DrugAge database) coexist with substantial biological noise.

Experimental validation demonstrated remarkable efficacy: Screening the top 0.07% of candidates identified by ElixirSeeker yielded four lifespan‐extending compounds in *C. elegans* from six tested—an over 50% success rate surpassing conventional screening yields. To promote open innovation, we provide ElixirFP, a modular Python package implementing our fingerprint fusion architecture. This resource enables customizable implementation of ensemble learning‐based fusion fingerprints across therapeutic domains, particularly benefiting research areas constrained by sparse and noisy biological data.

## 2 Materials and Methods

### 2.1 Data Sources and Pre‐Processing

Our study leveraged data from the DrugAge database (Barardo, Thornton, et al. 2017), which comprehensively catalogs small molecules with lifespan‐extending properties. DrugAge aggregates information on compounds, drugs, and supplements known to extend the lifespan of various model organisms, predominantly *C. elegans* , mice, and fruit flies. This database provides detailed aging‐related metrics, including average/median lifespan, maximum lifespan, strains, dosage, and gender, under standardized experimental conditions. The dataset is curated, incorporating data from diverse sources and rigorously controlled lifespan experiments. Given the substantial representation of compounds tested on *C. elegans* , our analysis primarily focused on this model organism. Additionally, we supplemented our dataset with information extracted from numerous academic publications (Büchter et al. 2013; Cheng et al. 2023; Cho et al. 2023; Ding et al. 2017; Havermann et al. 2014; Liu et al. 2022; Lu et al. 2017; Zhang et al. 2024; Yu et al. 2024; Zhao et al. 2017), resulting in a dataset comprising 1695 small molecules, including 462 positive instances.

In the context of lifespan‐extending drug screening, the problem can be abstracted into a mathematical framework, specifically as a binary classification problem. Here, the objective is to classify each molecule as either positive (having lifespan‐extending effects) or negative (lacking lifespan‐extending effects). Mathematically, this can be formulated using a binary label $y_{i}$ for each molecule i, where:

$$\left\{\begin{array}{c} 1\;\text{if molecule}\;i\;\mathrm{has}\;\text{anti}-\text{aging effects}\\ 0\;\text{if molecule}\;i\;\text{does not have anti}-\text{aging effects} \end{array}\right.$$

### 2.2 Feature Selection

#### 2.2.1 Generation for Compounds' Fingerprints

To obtain structural information of compounds, we first used the PubchemPy tool to extract the SMILES strings of all compounds from the DrugAge database (Southern and Griffin 2011). Using Python packages PubChemPy and Openbabel (O'Boyle et al. 2011), the chemical structures of the DrugAge dataset were converted into canonical SMILES strings.

Before fusing molecular fingerprints, we conducted pre‐training to determine the optimal lengths of three types of fingerprints: Morgan, Topological, and MACCS. We employed Python 3.7.10 and the following packages for generating fingerprints and training: catboost (version 1.2.5), joblib (version 1.3.2), lightgbm (version 4.3.0), numpy (version 1.22.0), pandas (version 2.0.3), rdkit (version 2023.9.5), rdkit‐pypi (version 2023.9.5), requests (version 2.31.0), scipy (version 1.10.1), scikit‐learn (version 1.3.2) and xgboost (version 2.0.3).

The Morgan fingerprint, also known as circular or extended connectivity fingerprint (ECFP), captures local structural features through iterative extension from each atom within a specified radius. It generates a binary fingerprint indicating the presence or absence of specific motifs, facilitating robust similarity searching and clustering. In contrast, the topological fingerprint, or path‐based fingerprint, encodes molecular structures by representing topological paths or fragments as binary substructure patterns. It adeptly captures both global and local structural nuances, particularly in delineating structural similarities and pharmacophoric landscapes.

The MACCS fingerprint, derived from the Molecular ACCess System (MACCS), adopts a fixed‐length representation based on predefined structural keys or pharmacophoric patterns. By identifying the presence or absence of each key, it yields a concise yet informative fingerprint.

These three types of fingerprints are calculated from different perspectives: the Morgan fingerprint captures local structural features through iterative extension, the topological fingerprint encodes molecular structures based on topological paths or fragments, and the MACCS fingerprint adopts predefined structural keys or pharmacophoric patterns for representation.

#### 2.2.2 Pre‐Training for Optimal Fingerprint Lengths

XGBoost (eXtreme Gradient Boosting) is a machine learning algorithm that is particularly suitable for solving classification and regression problems. Its core idea is to combine multiple simple models (usually decision trees) to form a more powerful model through “ensemble learning”. The working principle of XGBoost can be likened to “teamwork”: each simple model (decision tree) focuses on solving a part of the problem, while XGBoost continuously adjusts the weight of each model to ensure that they can work together and ultimately produce more accurate prediction results.

One key advantage of XGBoost is its ability to provide feature importance scores, which quantify the contribution of each feature to the model's predictive performance. Feature importance scores are calculated based on how frequently each feature is used in decision tree splits and how much each split improves the model's performance. Features that are frequently used in important splits and lead to significant improvements in model performance are assigned higher importance scores.

We utilized the XGBoost algorithm for this task. Prior to feature fusion, we conducted pre‐training to determine the optimal lengths of three different types of molecular fingerprints: Morgan, Topological, and MACCS. We enumerated fingerprint lengths from 16 to 1016 bits with increments of 8 (excluding MACCS), utilizing the XGBoost algorithm and employing ten‐fold cross‐validation to obtain the optimal length for each fingerprint type. Feature importance scores were recorded for each fingerprint at its optimal length.

### 2.3 Fusion of Molecular Fingerprints

After obtaining feature importance scores from pre‐training, we implemented an attention‐driven fusion approach to integrate the fingerprints effectively. For the detailed information on the algorithm, please refer to Appendix S1.

We used Kernel Principal Component Analysis (KPCA) with the Gaussian Radial Basis Function (RBF) kernel, leveraging the feature importance scores as weights in the KPCA. KPCA is a technique in machine learning that extends the traditional PCA by using kernel functions to map data into a higher‐dimensional space where it becomes linearly separable. This method is particularly useful when dealing with complex, non‐linear relationships in data.

KPCA works by transforming the original data into a new coordinate system, where the axes (principal components) are aligned with the directions of maximum variance in the data. Unlike standard PCA, which is limited to linear transformations, KPCA employs kernel functions, such as the Gaussian RBF kernel, to capture non‐linear patterns. The Gaussian RBF kernel measures the similarity between data points based on their distance, allowing KPCA to uncover intricate structures that would be missed by linear methods.

One of the key advantages of KPCA is its ability to incorporate feature importance scores as weights during the dimensionality reduction process. This weighted approach ensures that the principal components are more heavily influenced by the features that contribute most significantly to the predictive power of the model. By assigning higher weights to more important features, KPCA can prioritize the most relevant aspects of the data, leading to more meaningful and interpretable results.

This weighted KPCA approach is particularly beneficial in biological research, where datasets often contain a large number of features, many of which may be irrelevant or redundant. The RBF kernel used in KPCA measures the similarity between data points in the input space. The RBF kernel function is defined as:

(1) $$K\left(x_{i},x_{j}\right)=\exp\left(-\gamma \sum_{k=1}^{d}\omega_{k}{\left|x_{\mathrm{ik}}-x_{\mathrm{jk}}\right|}^{2}\right)$$

In this context, $x_{\mathrm{ik}}$ and $x_{\mathrm{jk}}$ represent the values of molecule *i* at the *k*‐th fingerprint bit, respectively. d is the total length of the fingerprint, and γ is the bandwidth parameter of the kernel, which controls the smoothness of the Gaussian function. ω is the feature importance score of the fingerprint bit, used to amplify the differences between the fingerprint bits. The architecture design was detailed in Appendix S1.

### 2.4 Model Validation

To ensure the robustness of the model, we employed a ten‐fold cross‐validation method. Throughout the machine learning process, the dataset was split into a ratio of 2:7:1, where 70% was allocated for training, 20% for validation, and the remaining 10% for testing. A ten‐fold cross‐validation was performed within the training set. Model performance was evaluated by the AUC score, with cross‐validation repeated 10 times to produce 10 AUC scores.

The predictive accuracy we report is based on the average AUC values from the ten‐fold cross‐validation and the cumulative confusion matrix. All models were scored with metrics of classification performance:

(2) $$\text{Accuracy}=\frac{\mathrm{TP}+\mathrm{TN}}{\mathrm{TP}+\mathrm{TN}+\mathrm{FP}+\mathrm{FN}}$$

where TP represents the count of true positives, TN denotes the count of true negatives, FP is the number of false positives, and FN stands for the number of false negatives.

Accuracy measures the proportion of correctly predicted instances (both true positives and true negatives) out of the total instances. It provides a general overview of the model's performance but can be misleading in imbalanced datasets.

(3) $$\text{Precision}=\frac{\mathrm{TP}}{\mathrm{TP}+\mathrm{FP}}$$

Precision indicates the proportion of correctly predicted positive instances (true positives) out of all instances predicted as positive. It is particularly useful when the cost of false positives is high.

(4) $$\text{Recall}=\frac{\mathrm{TP}}{\mathrm{TP}+\mathrm{FN}}$$

Recall (Sensitivity) represents the proportion of correctly predicted positive instances (true positives) out of all actual positive instances. It is crucial when the cost of false negatives is high, such as in medical diagnoses.

(5) $$F1-\text{Score}=2\cdot \frac{\text{Precision}\cdot \text{Recall}}{\text{Precision}+\text{Recall}}$$

F1‐Score combines precision and recall into a single metric by taking their harmonic mean. It is especially useful for evaluating models on imbalanced datasets, as it balances the trade‐off between precision and recall. Precision measures the proportion of correctly predicted positive instances out of all instances predicted as positive, while recall measures the proportion of correctly predicted positive instances out of all actual positive instances. In many real‐world scenarios, especially in biological research, datasets are often imbalanced, meaning one class significantly outnumbers the other. In such cases, relying solely on accuracy can be misleading, as a model might achieve high accuracy by simply predicting the majority class.

The F1‐Score addresses this issue by providing a single metric that considers both precision and recall, ensuring that the model performs well in identifying both positive and negative instances.

(6) $$\mathrm{AUC}-\mathrm{ROC}=\int_{0}^{1}\mathrm{TPR}\left(\mathrm{FPR}\right)\;d\left(\mathrm{FPR}\right)$$

AUC‐ROC (Area Under the Receiver Operating Characteristic Curve) measures the model's ability to distinguish between classes across all classification thresholds. A higher AUC value indicates better performance, with 1 representing perfect classification and 0.5 representing random guessing.

### 2.5 Molecular Fingerprint Stability Index

The Molecular Fingerprint Stability Index (MFSI) is a comprehensive metric utilized to assess the consistency of model performance across various molecular fingerprint lengths. This index is derived from the standard deviations of the model's accuracy, Area Under the Curve (AUC), and F1‐Score under different molecular fingerprint lengths, normalized to quantify the stability of model performance. MFSI ranges between 0 and 1, where values approaching 0 indicate minimal performance discrepancies across diverse molecular fingerprint lengths, thus indicating higher stability.

(7) $$\text{MFSI}=1-\frac{\sigma_{\mathrm{Acc}}+\sigma_{\mathrm{AUC}}+\sigma_{F1}}{3*\max\left(\sigma_{\mathrm{Acc}},\sigma_{\mathrm{AUC}},\sigma_{F1}\right)}$$

### 2.6 *C. elegans* Strains and Maintenance

In all experiments, the N2 strain was used as the wild type. Worms were maintained on NGM (Nematode Growth Medium) plates at 20°C, with the medium composed of 25 mM NaCl, 1.7% agar, 2.5 mg/mL peptone, 5 μg/mL cholesterol, 1 mM CaCl_2_, 1 mM MgSO_4_, and 50 mM KH_2_PO_4_ at pH 6.0.

Additionally, UV‐killed (dead) *Escherichia coli* strain OP50 was used as the food source in all experiments to ensure consistent experimental conditions and avoid potential confounding effects from bacterial metabolism or growth. To prepare the UV‐killed OP50, the concentrated bacterial suspension was spread onto prepared NGM plates. After the bacterial lawn had dried slightly, the plates were opened (lids removed) and placed directly into a UV crosslinker for irradiation. The energy was set to 9999 × 100 μJ/cm^2^, and the irradiation time was 12 min.

For synchronization, gravid adult worms were washed from plates using M9 buffer (22 mM KH2PO4, 42 mM Na2HPO4, 86 mM NaCl, 1 mM MgSO4) and treated with a hypochlorite solution (1% sodium hypochlorite, 0.5 M NaOH) for 10 min with gentle agitation to dissolve adult carcasses and release eggs. Eggs were pelleted by centrifugation at 1000 × *g* for 1 min, washed three times with M9 buffer, and hatched overnight in M9 buffer at 20°C to obtain synchronized L1 larvae. L1 larvae were then transferred to NGM plates seeded with OP50 and allowed to develop to the desired stage at 20°C.

### 2.7 Lifespan Assay

Solid drug powders were dissolved in DMSO to prepare 10 mM stock solutions. For each drug, the final DMSO concentration in the Nematode Growth Media (NGM) was adjusted based on solubility and biocompatibility. Medrysone, Thymoquinone, and Praeruptorin C were tested with 0.1% DMSO (v/v) in the medium. 7β‐Hydroxylathyrol, Polyphyllin VI, and α‐Hederin required 1% DMSO (v/v) for optimal solubility while maintaining viability. Control groups for all drug treatments were maintained in parallel under identical conditions, with NGM supplemented with the corresponding concentration of DMSO solvent but without the test compound.

The prepared drug stock was then proportionally added to the NGM to create solutions with concentrations of 25, 50, and 100 μM. Prior to all experiments, we had ensured that DMSO in selected concentrations did not affect worms in terms of longevity compared to vehicle‐free conditions (detailed in Figures S1 and S2; Tables S3–S6), ensuring its suitability as a solvent.

Lifespan assays were conducted at 20°C with daily scoring of survival under a stereomicroscope. Worms (*n* = 60–80 per group) were transferred to fresh plates every 2 days to avoid bacterial depletion. Worms lost due to desiccation or mechanical damage were censored from the analysis. Notably, no antibiotics or 5‐fluoro‐2′‐deoxyuridine (FUDR) were used throughout the experiments.

Worms confirmed as dead were removed from the medium, and the number of dead and lost worms in each culture dish was recorded. Worms lost due to crawling on the walls or handling errors, leading to non‐natural death, were counted as missing.

The lifespan of N2 worms was analyzed using the Kaplan–Meier survival curve method, which is a non‐parametric statistic used to estimate the survival function from lifetime data. To compare the survival curves between different groups, the log‐rank test was performed. This test assesses whether there are statistically significant differences in the survival distributions of the groups. A *p* value < 0.05 was considered statistically significant.

### 2.8 Heat Shock Stress Resistance Assay

Slimilar to lifespan assay, age‐synchronized worms were obtained by hypochlorite treatment of gravid adults. Synchronized eggs were cultured on NGM plates seeded with OP50 at 20°C until reaching the young adult stage (approximately 3 days post‐L1). Young adult stage worms were transferred to drug‐containing NGM plates (*n* = 60–80 per group, 15 worms per plate, 3 independent biological replicates were performed.) and maintained at 20°C for 5 days.

On Day 5, worms were subjected to heat shock stress by shifting plates to a 35°C incubator (0 h timepoint). Mortality was assessed every 2 h under a stereomicroscope. Worms displaying no response to gentle platinum wire touch were scored as dead.

Worms lost due to non‐natural causes (e.g., crawling on plate walls, desiccation artifacts, or handling errors) were censored at the time of disappearance and excluded from survival analysis (< 5% of total population in all groups). Survival curves were generated using the Kaplan–Meier method, and statistical significance was determined by the log‐rank test (Prism v9.0; *p* < 0.05 considered significant).

### 2.9 Statistical Analysis

All statistical analyses were performed using GraphPad Prism 10.1.0 (GraphPad Software, San Diego, CA, USA). A *p* value of < 0.05 was considered statistically significant. Data are presented as mean ± standard error of the mean (SEM). Survival analysis was conducted using the Kaplan–Meier (KM) method, and differences between survival curves were assessed using the log‐rank test.

## 3 Results and Discussion

### 3.1 The Fusion Fingerprint Significantly Enhanced Model Accuracy

Our study uses an expanded DrugAge database containing lifespan‐extending compounds validated across multiple species ( *C. elegans* > 70%) and incorporating newly reported molecules, resulting in a dataset of 1695 small molecules (462 positives, 1233 negatives).

Molecular fingerprints serve as computational representations that translate chemical structures into numerical vectors, enabling machine learning models to “interpret” molecular features. While fingerprints like Morgan (atom environment‐based), Topological (bond path‐based), and MACCS (substructure‐based) encode distinct aspects of molecular identity, each has inherent limitations in comprehensively capturing bioactive determinants.

Therefore, for machine learning models, the selection and design of molecular fingerprints directly determine the model's feature capture capabilities, which in turn affects its prediction results. Although different molecular fingerprints can encode certain specific aspects of the molecular structure, none of them can fully capture the multidimensional characteristics of molecules in complex biological activities.

For example, Morgan fingerprints may ignore the global topological information of molecules, while MACCS fingerprints may not be able to effectively identify novel molecular skeleton variants. These limitations are particularly prominent in small data sets (such as the lifespan‐extending compound database in this research) because the lack of data will amplify the incompleteness of feature capture, making it difficult for the model to learn enough information to accurately predict complex biological activities.

In our study, we conducted an investigation of the three distinct molecular fingerprinting methodologies as illustrated above. To establish a baseline, we employed the top‐performing single molecule fingerprint models.

The length of molecular descriptors can significantly impact prediction accuracy. To identify the optimal lengths for two variable‐length molecular fingerprints (Morgan and Topological fingerprints) that best suit our dataset, we evaluated models using these fingerprints across a range of bit lengths from 16 to 1016 bits. As shown in Figure 3A,B, the Morgan fingerprint achieved its best performance at 368 bits (prediction accuracy = 0.662), while the Topological fingerprint performed optimally at 696 bits (prediction accuracy = 0.701).

However, even after optimizing these fingerprints individually, their limitations became apparent. These optimized descriptors alone could not fully capture the chemical diversity of lifespan‐extending molecules, as their prediction accuracies remained below the desired threshold of 0.80. This suggests that no single molecular descriptor alone could comprehensively represent the structural and physicochemical features critical to lifespan‐extending activity. This needed multi‐fingerprint fusion to synergize their complementary strengths—local atomic detail of Morgan, global connectivity of Topological, and pharmacophoric motifs of MACCS.

Then we first applied principal component analysis (PCA) to the three optimized fingerprints (368‐bit Morgan, 696‐bit Topological, and MACCS). This fusion strategy, termed ElixirFP, aimed to capture complementary structural and physicochemical patterns while reducing feature redundancy. By enumerating lengths through grid search, we identified 192 bits as the optimal dimensionality for ElixirFP, achieving higher model efficiency (Table 1, Accuracy: 0.779 ± 0.033).

**TABLE 1:** Performance metrics of different molecular fingerprints at optimal lengths. This table summarizes the predictive performance of various fingerprint types (Morgan, Topological, MACCS, PCA, and Kernel‐PCA) at their best lengths, evaluated based on accuracy and ROC AUC.

Fingerprint	Length	Accuracy	ROC AUC
Morgan	368	0.727 ± 0.035	0.662 ± 0.029
Topological	696	0.746 ± 0.010	0.701 ± 0.031
MACCS	166	0.711 ± 0.026	0.639 ± 0.027
ElixirFP	192	0.779 ± 0.033	0.706 ± 0.058
Attention‐ElixirFP	**64**	**0.849 ± 0.012 (*p* < 0.001)**	**0.767 ± 0.020 (*p* < 0.001)**

*Note:* The bold row indicates the best performing method with statistically significant superiority to ElixirFP on paired t‐tests.

However, conventional PCA does not account for the fact that individual bits in molecular fingerprints may contribute differently to predicting lifespan‐extending potential. Hence, we used feature importance scores generated through XGBoost training as biologically weighted parameters to prioritize the most predictive molecular features.

These scores reflect the significance of each feature bit in predicting lifespan‐extending activity and were used to guide a modified PCA method (Kernel PCA, KPCA), enabling nonlinear dimensionality reduction that prioritizes features with the highest biological relevance. This approach is similar to the “attention mechanism” in deep learning, enabling the model to focus on the most critical features, and thus the resulting representation is termed Attention‐ElixirFP.

This attention‐driven method demonstrated higher compression efficiency. A 64‐bit Attention‐ElixirFP achieved higher performance metrics (Accuracy: 0.849 ± 0.012; ROC AUC: 0.767 ± 0.020), surpassing both single fingerprints and standard PCA‐fused ElixirFP as mentioned above (Table 1).

The Molecular Fingerprint Stability Index (MFSI) is a metric designed to evaluate the performance consistency (robustness) of molecular fingerprints across varying bit‐length configurations. Since conventional training workflows typically do not systematically test all possible fingerprint lengths, minimizing performance fluctuations across different bit‐length settings can enhance computational efficiency and streamline model optimization. As shown in Figure 3C, the Topological fingerprint exhibited the highest MFSI, indicating the largest performance variation across different bit‐length configurations. In contrast, Attention‐ElixirFP demonstrated the best stability in its performance across varying bit‐length settings.

This fusion framework introduces three key elements through context‐aware weighting, non‐linear feature synthesis, and dimensionality‐efficiency optimization. By using XGBoost's feature importance scores as biologically informed weights, Elixieseeker prioritizes molecular descriptors that are most relevant to lifespan‐extending related phenotypes, establishing a chemically interpretable weighting scheme.

### 3.2 Critical MACCS Fragments Driving Lifespan‐Extending Activity

While our phenotype‐based drug discovery approach enables rapid identification of lifespan‐extending compounds, this strategy inherently carries limitations that warrant interpretation. Firstly, the de novo nature of PDD leads to a dissociation phenomenon, as our models effectively map structural patterns to phenotypic outcomes, such as extending lifespan, while remaining agnostic to the underlying biological targets. This is similar to identifying keys that fit a lock without understanding the lock's internal mechanism, as evidenced by recent critiques of deep learning in drug discovery.

Thus, identification of key molecular fragments represents an important step in developing potent lifespan‐extending compounds. The MACCS (Molecular Access System) fingerprint, an established molecular descriptor comprising 166 predefined substructural patterns, serves as a tool for encoding structural information of small molecules. This fingerprinting system has demonstrated extensive utility in drug discovery and cheminformatics applications, particularly for establishing structure–activity relationships.

**FIGURE 2:**
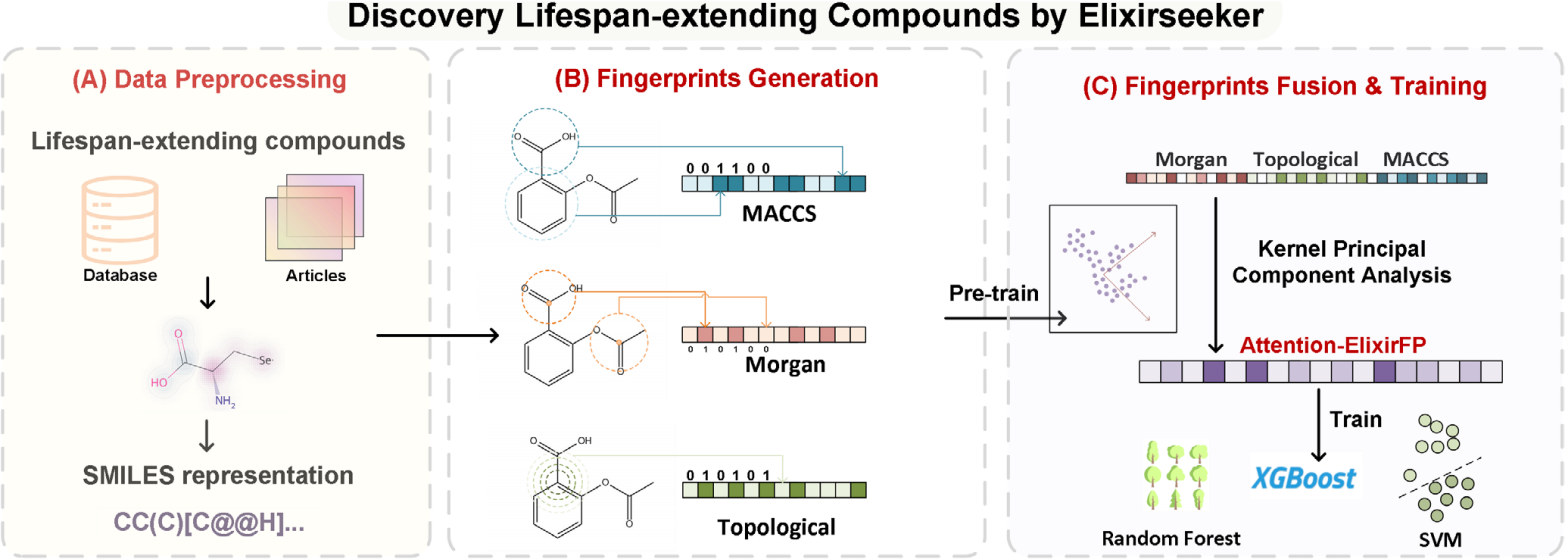
An overview of ElixirSeeker.

Our analysis revealed two MACCS indices with significance: index 161 (importance score = 11.41) and index 26 (importance score = 3.28). As illustrated in Figure 2F, index 161 corresponds to the presence of nitrogen atoms, while index 26 characterizes carbon atoms participating in cyclic systems with specific double bond configurations and connectivity patterns. These structural elements emerged as critical determinants in predictive models for lifespan‐extending activity.

For carbon atoms involved in specific double bond configurations and connection patterns, especially carbon–carbon double bonds in ring systems, fullerenes C60 contain multiple carbon–carbon double bonds and are thought to extend lifespan by scavenging free radicals. In addition, resveratrol and curcumin have been widely studied for their antioxidant and anti‐inflammatory effects due to their conjugated double‐bond system, and have been used for lifespan‐extending purposes.

Notably, nitrogen‐containing fragments dominated the top‐ranked structural motifs, constituting two of the five most significant features (Table 2). This observation underscores nitrogen's role in bioactive molecule design, particularly in distinguishing active compounds from inactive counterparts. The chemical versatility of nitrogen—manifested through its ability to form diverse bonding configurations (e.g., amines, amides, and imines)—establishes it as a fundamental component in biologically active molecular architectures. These structural revelations may guide pharmacophore optimization: (1) preferential introduction of pyridine nitrogen (similar to metformin) in the benzene ring neighborhood to balance lipophilicity; (2) introduction of a sulfonyl group in the double bond neighborhood to construct a redox‐responsive switch; and (3) substitution of the methylene bridge for an oxoheteroatom may mimic the endogenous anti‐aging conformation of urushiolic acid.

**TABLE 2:** Key structural motifs identified using MACCS (Molecular ACCess System) indices. This table presents the MACCS indices with the highest importance scores, along with their corresponding SMARTS notations and descriptions of the molecular structures they represent. The indices illustrate crucial chemical features that significantly contribute to the lifespan‐extending properties of compounds.

MACCS Index	Score	Description
161	11.411	Nitrogen atom
26	3.238	Structure of a carbon–carbon double bond (C=C) between two carbon atoms
40	1.526	Sulfur‐oxygen single bond
147	1.495	A methylene bridge (—CH_2_—CH_2_—) linked to any other atom
25	0.964	Structure of a carbon atom directly connected to three nitrogen atoms

However, it is important to note the inherent limitations of MACCS fingerprinting: its predefined 167 patterns may miss three‐dimensional pharmacophores that are unique to novel anti‐aging drugs. For example, the planar carboxylic acid structure of α‐ketoglutarate analogues is not captured by specific MACCS sites.

### 3.3 The 64‐Bit Attention‐ElixirFP Achieves Optimal Performance

During the KPCA dimensionality reduction process, we observed that both 64‐bit and 624‐bit Attention‐ElixirFP achieved peak performance. Notably, the 64‐bit variant demonstrated exceptional efficiency, outperforming the 192‐bit PCA‐fused ElixirFP (Accuracy: 0.849 vs. 0.779; ROC AUC: 0.767 vs. 0.706, Table 1) despite its significantly compressed feature length (only ~10% of the original 696‐bit Morgan fingerprint). This highlights KPCA's ability to effectively distill lifespan‐extending molecular signatures by converting sparse binary representations into dense floating‐point vectors enriched with discriminative chemical patterns.

However, to fully validate the generalizability of Attention‐ElixirFP beyond XGBoost frameworks, we evaluated three variants—192‐bit ElixirFP, 624‐bit Attention‐ElixirFP, and 64‐bit Attention‐ElixirFP—across 11 machine learning classifiers (including Decision Trees, Random Forests, and ExtraTrees) paired with seven sampling strategies (e.g., SMOTE, RandomOverSampling). Given the class imbalance inherent in lifespan‐extending datasets, we prioritized F1‐Score as the evaluation metric—a harmonic mean of precision (correct identification of potential hits) and recall (comprehensive detection of all true actives) that ensures reliable prediction of bioactive compounds. This balanced measure better reflects real‐world screening scenarios where both minimizing false leads and maximizing hit discovery are critical.

**FIGURE 3:**
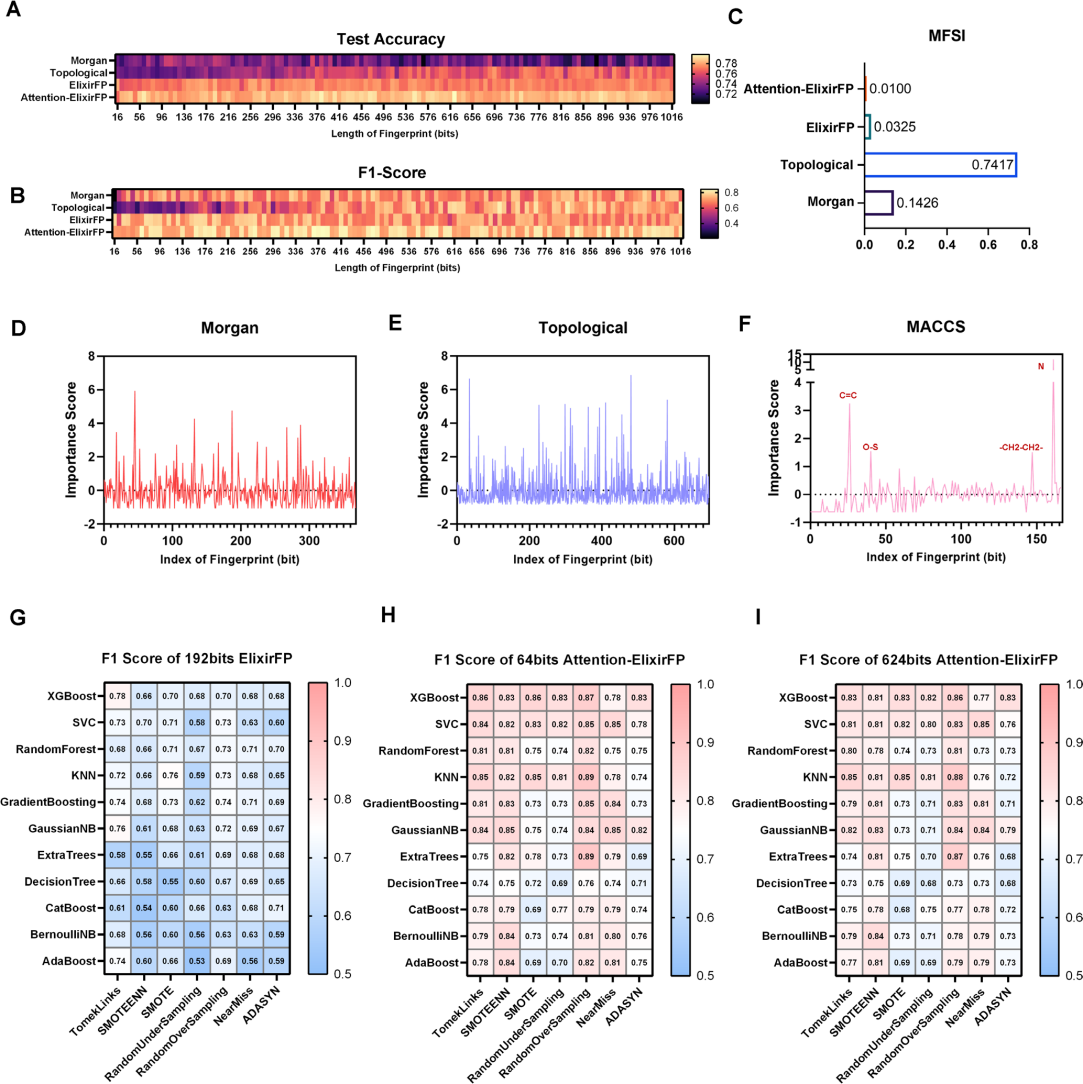
Performance and analysis of the Elixirseeker model. (A) Accuracy heatmaps of XGBoost models trained with Morgan, Topological, ElixirFP, and Attention‐ElixirFP fingerprints on the test set (across fingerprint lengths from 16 to 1016 bits). (B) F1‐Score heatmaps of the same XGBoost models on the test set. (C) Molecular Fingerprint Stability Index (MFSI) for Morgan, Topological, ElixirFP, and Attention‐ElixirFP. (D–F) Feature importance scores for individual bits of Morgan (D), Topological (E), and MACCS (F) fingerprints. (G–I) F1‐Scores of machine learning models using 192‐bit ElixirFP (G), 64‐bit Attention‐ElixirFP (H), and 624‐bit Attention‐ElixirFP (I) across different algorithms and sampling methods.

As shown in the heatmaps (Figure 3G–I), the 192‐bit ElixirFP demonstrated mostly lower F1‐Scores across all model‐sampling combinations compared to Attention‐ElixirFP variants, with the 64‐bit Attention‐ElixirFP consistently outperforming its 624‐bit counterpart. The “ExtraTrees + RandomOverSampling” configuration achieved the highest F1‐Score (0.892 ± 0.014), likely due to ExtraTrees' inherent feature randomness mitigating overfitting and RandomOverSampling's effectiveness in addressing minority class underrepresentation. In contrast, ElixirFP showed larger performance variability across classifiers, underscoring the limitations of linear feature aggregation for complex phenotypic modeling.

This finding carries significant implications for molecular machine learning. Traditional fingerprints often impose computational bottlenecks due to their high dimensionality (e.g., 1230 bits when concatenating Morgan, Topological, and MACCS). Our attention‐driven compression strategy reduces feature length by 84% while enhancing predictive power, effectively creating a task‐specific “lifespan‐extending fingerprint” that concentrates bioactive patterns from training molecules. Such compact representations dramatically lower computational costs for large‐scale virtual screening, reducing training time by 63% compared to using raw concatenated fingerprints (from 126 min to 45 min per 10‐fold cross‐validation).

Moreover, in scenarios with sparse biological data (common in aging research), the 64‐bit Attention‐ElixirFP maximizes feature extraction efficiency, minimizing noise from redundant or irrelevant molecular descriptors. These findings may help address longstanding challenges in phenotype‐based drug discovery, where computational efficiency and biological interpretability must coexist by enabling high‐accuracy predictions with ultra‐compact representations.

### 3.4 Candidate Compounds Enhance *C. elegans* Survival Under Heat Stress

After optimizing and integrating molecular fingerprints, we expanded the application of our model to an external compound database to assess its generalizability and effectiveness outside the initial training set. Using the 64‐bit Attention‐ElixirFP developed in this study, our screening process yielded notable results, particularly with the “ExtraTrees + RandomOverSampling” ensemble method as the final ElixirSeeker Model. Notably, we identified several potential compounds with high prediction confidence.

**FIGURE 4:**
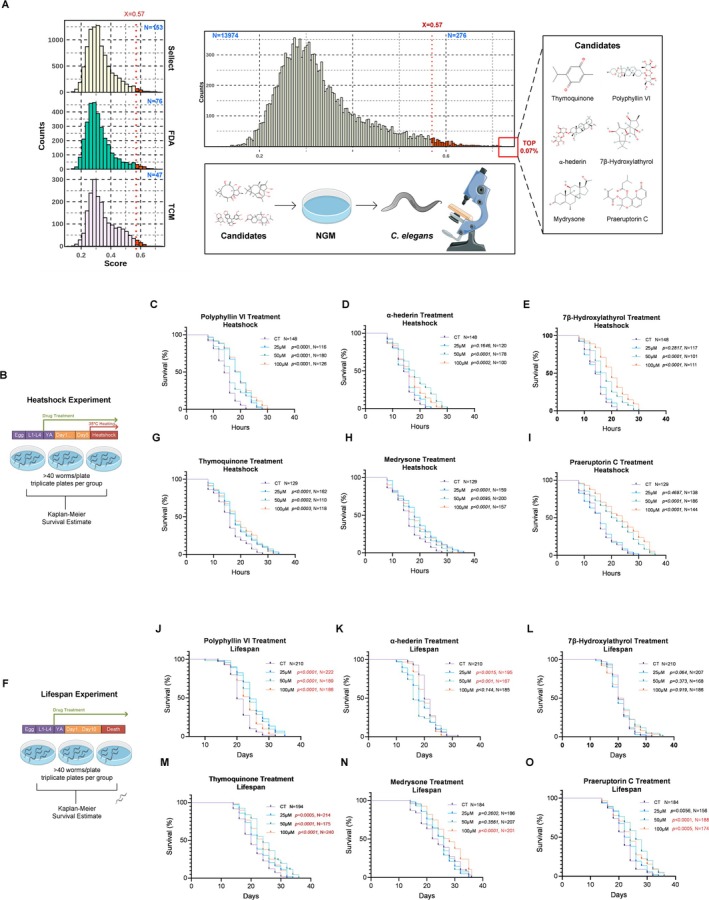
External database screening and validation of candidate compounds in *C. elegans* . (A) Left: Density distribution plots of prediction scores for three compound libraries (FDA‐approved: Green, Sellect Bioactive: Yellow, Traditional Chinese Medicine: Pink). Middle: Aggregated density distribution of all screened compounds (*n* = 13,974), with the top 2% threshold (score > 0.57) marked by a dashed line. Right: Chemical structures of six prioritized candidates. (B) Schematic of heat shock assay protocol. Synchronized young adult nematodes were treated with compounds until Day 5 post‐synchronization, followed by acute thermal stress at 35°C until mortality. (C–E, G–I) Kaplan–Meier survival curves under heat shock for (C) Polyphyllin VI, (D) α‐Hederin, (E) 7β‐Hydroxylathyrol, (H) Thymoquinone, (I) Medrysone, and (J) Praeruptorin C. *p* values derived from log‐rank test; N indicates sample size per group. (F) Schematic of lifespan assay. Young adult nematodes were continuously exposed to compounds until natural death. (J–O) Kaplan–Meier lifespan curves for (J) Polyphyllin VI, (K) α‐Hederin, (L) 7β‐Hydroxylathyrol, (M) Thymoquinone, (N) Medrysone, and (O) Praeruptorin C. *p* values calculated by log‐rank test; Censored data (lost animals) are excluded from analysis. The control groups (CT) shown in the figures were treated with DMSO solvent at concentrations identical to those in the corresponding experimental groups.

Our screening process included three compound libraries: the FDA approved library (TargetMol, USA, L1010), the Sellect Bioactive compound library (Selleckchem, Houston, TX, USA, L1700), and a Traditional Chinese Medicine compound library (Chengdu Biopurify Phytochemicals Ltd.), which contained 3151, 9109, and 1988 small molecules, respectively. Using our model for prediction, we obtained a maximum score of 0.716. We then selected the top 50 small molecules with a threshold score of 0.57 (Figure 4A).

Most compounds of the database were assigned prediction scores indicating a low likelihood of being senolytic‐like. Further analysis of the compounds over this threshold yielded intriguing findings. Some molecules had previously been validated as positive compounds, such as Triptolide (Hsu et al. 2012) and Ginkgolid B (Lee et al. 2025) which are known to extend the lifespan of model organisms, and they did not occur in the training set. Four of the top 15 compounds were known positive drugs, and we selected six of these compounds that were readily available for subsequent testing. The candidates were detailed in Table 3 and Table S44.

**TABLE 3:** Top 15 compounds screened by the Elixirseeker model. Compounds labeled as “Known anti‐aging” or “DrugAge positive” have been previously reported to have lifespan‐extending properties. The complete list of TOP50 compounds along with detailed descriptions of each drug can be found in Table S44.

Score	Name
0.716523	Praeruptorin C (this work)
0.708575	20‐Deoxyingenol
0.708465	Ginkgolid B (Known anti‐aging [Lee et al. 2025])
0.707907	Praeruptorin E
0.705013	Polyphyllin VI (this work)
0.704307	Etoposide
0.703291	Triptolide (DrugAge positive)
0.701729	Ginkgolid A
0.701113	NAD+ (DrugAge positive)
0.700947	Pregnenolone (DrugAge positive)
0.699309	Medrysone (this work)
0.69821	Alpha‐Hederin (this work)
0.697395	Lathyrol
0.696004	7‐beta‐Hydroxylathyrol (this work)
0.6956	Thymoquinone (this work)

The composition of small molecules with a score greater than the threshold in the Traditional Chinese Medicine compound database is particularly interesting. Notably, 89.36% of these compounds belong to the saponin class, which is known for its anti‐aging and lifespan‐extending properties. Specifically, 40% of these saponins are Ginsenosides, including Ginsenoside F1, showing some relevance of Ginseng in this research. Moreover, in the Traditional Chinese Medicine database, Thymoquinone emerged with a high score of 0.696, surpassing the second‐ranked Ginsenoside‐Rh4 (0.637). This prompted our interest in investigating whether Thymoquinone could extend the lifespan of *C. elegans* , hence revealing its potential as an anti‐aging compound. Although reports have suggested the activity of Thymoquinone, no study has demonstrated that it extends lifespan in any model organism.

Then, we conducted tests on a selection of top‐performing small molecules identified from the three libraries, which had not been previously reported to have lifespan‐extending activities. These molecules included Praeruptorin C, Polyphyllin VI, α‐Hederin, Medrysone, Thymoquinone, and 7β‐Hydroxylathyrol. These compounds were chosen based on their high prediction scores and their absence in the literature concerning direct lifespan‐extending or senolytic activities.

The tested concentrations (25, 50, 100 μM) were selected based on alignment with common *C. elegans* dosing ranges in DrugAge studies (typically 10–200 μM), and preliminary solubility assessments. Importantly, these values represent agar medium concentrations, not actual internal doses. Nematodes ingest compounds orally while grazing on bacteria, resulting in substantially lower bodily concentrations due to partial absorption and metabolic degradation. This pharmacokinetic disconnect complicates direct comparisons to in vitro IC50 values, which were not pursued here.

We first performed heat shock stress assays on *C. elegans* at 35°C (Figure 4B; see Section 2 for protocol details). While heat shock experiments provide rapid preliminary insights into stress resistance as a proxy biomarker correlated with aging mechanisms, all six tested compounds demonstrated lifespan‐prolonging trends under thermal stress (Figure 4C–E,G–I, detailed in Table S1). However, the uniform concentration protocol likely created suboptimal dosing conditions for certain molecules, as biological activity thresholds vary by compound. Crucially, this result itself confirms bioactive potential, suggesting either antioxidant properties or other cytoprotective mechanisms common to these candidate compounds.

Notably, lifespan extension under acute heat shock conditions reflects transient stress protection rather than authentic healthspan improvement. This distinction is critical: many bioactive molecules (e.g., polyphenols, HSP inducers) exhibit such protective effects without necessarily modifying fundamental aging processes (Kumsta and Hansen 2017). Our findings align with the limitations of the DrugAge database, wherein some of the reported longevity compounds exhibit context‐dependent effects or fail validation in standardized lifespan assays, primarily manifesting partial cytoprotective benefits rather than authentic healthspan extension. This inherent bias introduces some implications for machine learning approaches. This will be discussed later.

The four active compounds' chemical classification spans steroidal saponins, quinones, coumarins, and synthetic hormones. Thymoquinone, the principal bioactive quinone derivative from *Nigella sativa* seeds (Arabic: Habatul Barakah), has been historically employed in Arabian medicine for respiratory disorders and immune modulation (Othman et al. 2024). Its potent antioxidant properties have prompted investigation as both a therapeutic agent and natural food preservative. The distribution of these compounds on the PCA landscape, as well as the positive compounds similar to them, is shown in Figure S4.

Polyphyllin VI, a steroidal saponin isolated from Paris polyphylla (Chinese: Chonglou), has been used for millennia in East Asian traditional medicine for detoxification, anti‐inflammatory, and anti‐neoplastic purposes. Modern pharmacological studies reveal its broad‐spectrum antitumor activity through apoptosis induction and cell cycle regulation mechanisms (Teng et al. 2020).

Praeruptorin C, a coumarin derivative extracted from the traditional Chinese medicinal herb Peucedanum praeruptorum Dunn (Chinese: BaihuaQianhu), was first documented in the Shennong Bencao Jing (ca. 200 ce) for managing respiratory conditions. Some studies have revealed its potential in modulating inflammatory responses and oxidative stress (Li et al. 2023). Medrysone, a synthetic glucocorticoid, contrasts with the natural products through its engineered pharmacological profile (Zhu et al. 2023).

### 3.5 Candidate Compounds Extend the Lifespan in *C. elegans*

Then, we evaluated the longevity‐enhancing potential of six candidate compounds through standardized *C. elegans* lifespan assays (Figure 4F–O, detailed in Table S2). Four small molecules demonstrated statistically significant lifespan extension effects: Polyphyllin VI (25, 50, and 100 μM; *p* < 0.0001, *N* = 222, 189, and 186, respectively, Figure 4F), Thymoquinone (50 and 100 μM; *p* < 0.0001, *N* = 175 and 240, respectively, Figure 4M), Medrysone (100 μM; *p* < 0.0001 *N* = 201, see Figure 4N), and Praeruptorin C (50 μM: *p* = 0.0005, *N* = 174, 100 μM: *p* < 0.0001, *N* = 188, Figure 4O).

We observed distinct dose–response relationships between lifespan extension and heat shock resistance assays, where optimal concentrations frequently diverged (e.g., Thymoquinone: 100 μM for lifespan vs. 25 μM for thermotolerance). This likely arises from fundamental differences in the biological pathways engaged by each assay. Heat shock resistance predominantly reflects acute activation of stress‐responsive pathways, such as the HSF‐1‐mediated proteostasis network or SKN‐1/Nrf2‐driven antioxidant responses (Kumsta and Hansen 2017). These systems exhibit rapid, nonlinear activation thresholds, where even low compound concentrations may saturate critical signaling nodes (e.g., Keap1‐Nrf2 dissociation) to confer transient protection. In contrast, lifespan extension necessitates chronic modulation of core aging mechanisms—such as insulin/IGF‐1 signaling (IIS), mitochondrial homeostasis, or epigenetic regulation—which operate over longer timescales and require sustained, balanced pathway engagement. Higher doses may inadvertently perturb compensatory feedback loops or induce off‐target toxicity, as exemplified by the hormetic response curve of Nrf2 activators, where excessive dosing paradoxically elevates oxidative stress.

However, models trained on DrugAge data may preferentially identify compounds with strong stress‐shielding properties, while failing to adequately capture features critical for regulating fundamental aging pathways. This aligns with the limitations observed in earlier machine learning studies on the DrugAge database, where incomplete capture of aging‐related features often resulted in suboptimal performance in wet‐lab experiments (Bene and Salmon 2023; Barardo, Newby, et al. 2017). To address this issue, our Attention‐ElixirFP framework is designed to maximize the capture of lifespan‐extending‐related features. Furthermore, to validate our approach, we constructed a smaller training set comprising only well‐established longevity‐promoting compounds (e.g., metformin, rapamycin) and re‐evaluated our candidate molecules. The results showed that these candidates consistently ranked highly (detailed in Table S7).

### 3.6 Candidate Compounds Significantly Enhance Locomotor Capacity in *C. elegans*

To contextualize aging trajectories, we referenced conserved hallmarks of physiological decline (e.g., motility loss, pharyngeal pumping rate decrease) that define functional age equivalence between species (Wei et al. 2025). Additionally, WormCNN is a framework for assessing the age of *C. elegans* using computer vision to determine if the nematodes are younger by comparing their images with controls (Pan et al. 2024), and all four positive drugs from the lifespan experiment showed a trend towards more youthfulness, as detailed in Table S8.

**FIGURE 5:**
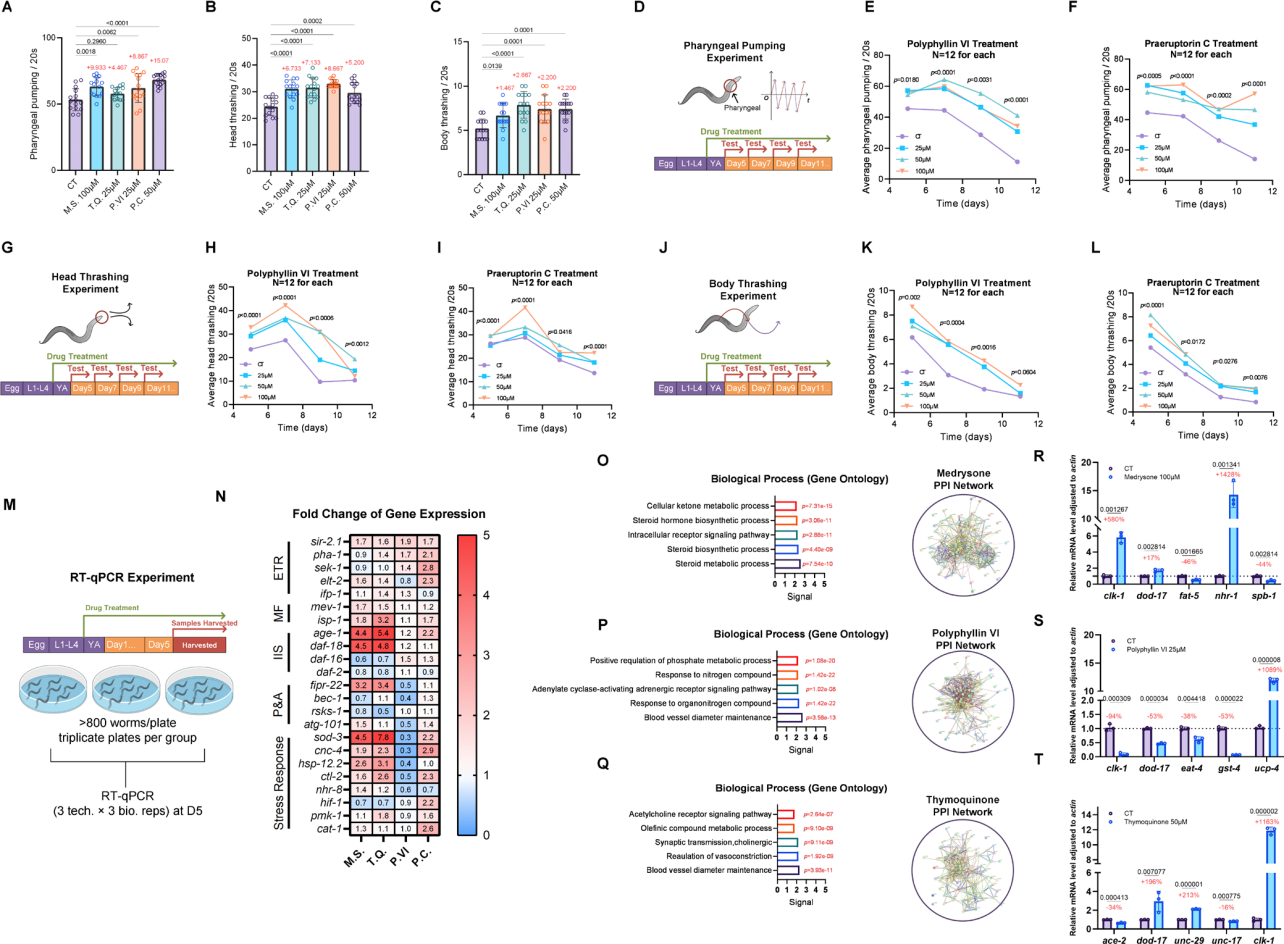
Behavioral phenotyping and mechanistic insights of candidate compounds in *C. elegans* . (A–C) Quantitative assessments of (A) pharyngeal pumping rate, (B) body bending frequency, and (C) head thrashing activity in control versus compound‐treated groups: 100 μM Medrysone (M.S.), 25 μM Thymoquinone (T.Q.), 25 μM Polyphyllin VI (P.VI), and 100 μM Praeruptorin C (P.C.). Bar heights represent mean ± SEM; red numerals denote mean difference relative to control (Dunnett's post hoc test, *p* < 0.05). (D) Schematic of pharyngeal pumping assay protocol: Synchronized Young Adult (YA) nematodes were treated with compounds and assessed at Days 5, 7, 9, and 11 post‐synchronization. (E, F) Time‐resolved pharyngeal pumping rates for (E) Polyphyllin VI (25, 50, 100 μM) and (F) Praeruptorin C (25, 50, 100 μM). *p* values reflect daily one‐way ANOVA. (full Dunnett's test results in Table S9, raw data see Tables S13–S20) (G) Schematic of head thrashing assay protocol, with assessments at Days 5, 7, 9, and 11. (H, I) Temporal dynamics of head thrashing activity for (H) Polyphyllin VI and (I) Praeruptorin C (full Dunnett's test results in Table S10, raw data see Tables S21–S28). *p* values reflect daily one‐way ANOVA. (J) Schematic of body bending assay protocol, performed at days 5, 7, 9, and 11. (K, L) Body bending frequency trajectories for (K) Polyphyllin VI and (L) Praeruptorin C *p* values reflect daily one‐way ANOVA. (full Dunnett's test results in Table S11, raw data see Tables S29–S36) (M) Experimental workflow for RT‐qPCR: RNA was extracted from Day 5 YA nematodes (*n* = 3 biological replicates per group, each containing 50–100 worms). (N) Heatmap of fold‐changes in aging‐related gene expression (vs. control) for M.S. (100 μM), T.Q. (25 μM), P.VI (25 μM), and P.C. (100 μM). Color scale: Red = upregulation, blue = downregulation. (O–Q) Network pharmacology analyses for (O) Medrysone, (P) Polyphyllin VI, and (Q) Thymoquinone: Left: Protein–protein interaction (PPI) networks of predicted targets; Right: Go biological process enrichment (top 5 terms). (R–T) RT‐qPCR validation of pathway‐specific gene expression for (R) Medrysone, (S) Polyphyllin VI, and (T) Thymoquinone. Data normalized to *C. elegans* Actin (mean ± SD; *p* < 0.05 by two‐tailed *t*‐test). All primers are detailed in Table S43.

As shown in Figure 5, we evaluated the effects of four lifespan‐extending candidates at their optimal concentrations (determined in prior assays) on three locomotor parameters: pharyngeal pumping, body bending, and head thrashing (Figure 5A–C). Statistical analyses revealed pronounced enhancements across these metrics (Table 4). Medrysone (100 μM) increased pharyngeal pumping by 9.93 contractions/min (*p*.adj = 0.0018), while Praeruptorin C (50 μM) demonstrated the most robust effect, elevating pumping rates by 15.07 contractions/min(*p*.adj < 0.0001). All compounds significantly improved body bending frequency (*p*.adj < 0.001), with Polyphyllin VI (25 μM) showing the greatest enhancement. Head thrashing activity was similarly augmented, particularly by Thymoquinone (25 μM) and Praeruptorin C (50 μM), both achieving ~2.67 additional thrashes/min (*p*.adj < 0.0001).

**TABLE 4:** Dunnett's multiple comparisons of locomotor parameters between control and compound‐treated groups.

Group	*N*	MD	95% CI of MD	*p*.adj	Sig.
CT vs. M.S. 100 μM (pharyngeal pumping)	15	−9.933	−16.72, −3.149	0.0018	Yes
CT vs. T.Q. 25 μM (pharyngeal pumping)	15	−4.467	−11.25, 2.317	0.2960	No
CT vs. P.VI 25 μM (pharyngeal pumping)	15	−8.867	−15.65, −2.083	0.0062	Yes
CT vs. P.C. 50 μM (pharyngeal pumping)	15	−15.07	−21.85, −8.283	< 0.0001	Yes
CT vs. M.S. 100 μM (body thrashing)	15	−6.733	−9.720, −3.747	< 0.0001	Yes
CT vs. T.Q. 25 μM (body thrashing)	15	−7.133	−10.12, −4.147	< 0.0001	Yes
CT vs. P.VI 25 μM (body thrashing)	15	−8.667	−11.65, −5.680	< 0.0001	Yes
CT vs. P.C. 50 μM (body thrashing)	15	−5.200	−8.186, −2.214	0.0002	Yes
CT vs. M.S. 100 μM (head thrashing)	15	−1.467	−2.694, −0.2392	0.0139	Yes
CT vs. T.Q. 25 μM (head thrashing)	15	−2.667	−3.894, −1.439	< 0.0001	Yes
CT vs. P.VI 25 μM (head thrashing)	15	−2.200	−3.427, −0.9726	0.0001	Yes
CT vs. P.C. 50 μM (head thrashing)	15	−2.200	−3.427, −0.9726	0.0001	Yes

Abbreviations: MD, Mean Difference; Sig, Significance.

Given their synergistic benefits in lifespan extension, thermotolerance, and acute locomotor enhancement, we prioritized Polyphyllin VI and Praeruptorin C for longitudinal behavioral profiling. Under our 20°C culture conditions, *C. elegan*s at days 5–11 post‐Young Adult stage operationally represent progressive functional aging stages, approximately mapping to human middle adulthood (Day 5, ~50 years) through advanced age (Day 11).

Pharyngeal pumping rates exhibited a biphasic trajectory, peaking at Day 7 (mid‐adulthood equivalent) before declining (Figure 5E,F, detailed in Table S9). Remarkably, Polyphyllin VI (100 μM) sustained a 400% increase in pumping activity at Day 11 (*p* < 0.0001 vs. control), while Praeruptorin C (100 μM)‐treated worms maintained youth‐like pumping capacity with no significant difference between Days 5 and 12 (*p* = 0.12). Head and body movement enhancements were predominantly observed during mid‐to‐elder adulthood (Days 5–9), suggesting distinct temporal mechanisms: sustained proteostasis/neuromuscular maintenance for pharyngeal function versus transient metabolic activation for somatic motility (detailed in Tables S10 and S11).

### 3.7 Mechanistic Profiling Reveals Divergent Pathway Activation by Candidate Compounds

To elucidate potential mechanisms underlying the observed geroprotective effects, we performed RT‐qPCR analyses on 23 aging‐related genes spanning five evolutionarily conserved pathways including epigenetic/transcriptional regulation (P&A), proteostasis/autophagy (ETR), mitochondrial function (MF), insulin/IGF‐1 signaling (IIS), and stress response. Synchronized young adult worms were treated with the best lifespan‐extending concentrations of Medrysone (100 μM), Thymoquinone (25 μM), Polyphyllin VI (25 μM), and Praeruptorin C (100 μM), followed by transcript quantification (Figure 5M,N). However, each compound exhibited distinct regulatory signatures despite shared phenotypic outcomes in longevity and locomotor enhancement. Using a threshold of |log2FC| ≥ 1 (*p* < 0.05), we delineated transcriptional signatures that mechanistically link to their geroprotective phenotypes.

Medrysone upregulated *sod‐3* (4.45‐fold), *age‐1* (4.38‐fold), *daf‐18* (4.49‐fold), *fipr‐22* (3.17‐fold), and *hsp‐12.2* (2.58‐fold), collectively indicating a dual antioxidant–proteostatic mechanism. The strong induction of *sod‐3* (mitochondrial SOD) and *hsp‐12.2* (chaperone) aligns with its thermotolerance enhancement (Figure 4A), while *age‐1* (PI3K) and *daf‐18* (PTEN) upregulation paradoxically amplifies IIS signaling—a phenomenon previously linked to DAF‐16‐independent longevity in *age‐1* gain‐of‐function mutants (Qi et al. 2021). The concomitant *fipr‐22* induction (ER stress regulator) suggests unfolded protein response (UPR) engagement, synergizing with HSP‐12.2 to maintain proteostasis under stress.

Thymoquinone activated stress (*sod‐3*:7.81‐fold; *ctl‐2*:2.63‐fold), mitochondrial (*isp‐1*:3.22‐fold; *cnc‐4*:2.34‐fold), and IIS pathways (*age‐1*:5.39‐fold; *daf‐18*:4.77‐fold), while suppressing *rsks‐1* (0.50‐fold, mTORC1 component). The *isp‐1* (complex III) and *cnc‐4* (Nrf2 homolog) upregulation implies mitochondrial redox optimization coupled with Nrf2‐mediated detoxification, rationalizing its superior locomotor enhancement (Figure 5C) via sustained ATP production. Paradoxically, *rsks‐1* downregulation (mTOR inhibition) contrasts with *age‐1* upregulation, suggesting a compensatory IIS‐mTOR crosstalk that transiently primes stress *resilience* without impairing autophagic flux (Pandey et al. 2024).

Polyphyllin VI uniquely suppressed antioxidant genes (*sod‐3*:0.33‐fold; *cnc‐4*:0.26‐fold; *hsp‐12.2*:0.36‐fold) and *sir‐2.1* (0.19‐fold) while upregulating *pha‐1* (1.70‐fold), a pharyngeal morphogenesis regulator. This “controlled oxidative priming” strategy—reducing baseline antioxidant reserves—likely activates FOXO/DAF‐16 (1.46‐fold) via hormesis, bypassing canonical IIS/sirtuin pathways (Kim and Webb 2017). The *pha‐1* induction correlates with its pharyngeal pumping maintenance in aged worms (Figure 5E), potentially stabilizing neuromuscular junction integrity (Schnabel et al. 1991).

Praeruptorin C upregulated mitochondrial (*hif‐1*:2.16‐fold; *cat‐1*:2.59‐fold), stress (*sek‐1*:2.76‐fold; *ctl‐2*:2.25‐fold), and IIS (*age‐1*:2.20‐fold; *elt‐2*:2.25‐fold) effectors. The *hif‐1* (hypoxia sensor) and *cat‐1* (catalase) induction points to HIF‐1α‐mediated metabolic reprogramming, favoring glycolysis while mitigating ROS via catalase—a strategy mirroring hypoxia‐induced longevity. Concurrent *sek‐1* (p38 MAPK) activation supports neuromuscular plasticity, explaining its sustained locomotion (Figure 5F). The *pha‐1* upregulation (2.13‐fold) synergizes with *elt‐2* (intestinal transcription factor) to stabilize pharyngeal‐intestinal signaling, an axis for systemic aging modulation (Schnabel et al. 1991; Su et al. 2020).

Despite mechanistic divergence, all compounds engage IIS (*age‐1/daf‐18* upregulation) and stress response pathways, albeit through distinct nodes: Medrysone and Thymoquinone amplify antioxidant defenses, while Polyphyllin VI and Praeruptorin C prioritize metabolic or structural adaptations. Crucially, *pha‐1* emerges as a pharyngeal aging biomarker, with both Polyphyllin VI and Praeruptorin C targeting its expression through disparate upstream regulators (*daf‐16* vs. *hif‐1*).

### 3.8 Network Pharmacology Analysis and Mechanistic Validation

Following the qPCR validation of aging‐related genes, target prediction serves as an essential next step to elucidate pharmacological mechanisms. Network pharmacology provides a systems‐level framework to map core responsive gene modules through protein–protein interaction (PPI) networks. We performed target profiling for the four active compounds and constructed protein–protein interaction (PPI) networks based on network pharmacology principles—a methodology that identifies core responsive genes by mapping their functional interconnectivity. Due to low target prediction confidence, Praeruptorin C was excluded from further analysis. For the remaining three compounds (Medrysone, Polyphyllin VI, and Thymoquinone), PPI network‐based GO enrichment analysis revealed distinct pathway associations as below (predicted targets detailed in Tables S39–S42).

Medrysone exhibited significant enrichment in steroid metabolic processes, aligning with its nuclear receptor *nhr‐1* overexpression (14.3‐fold, *p* = 0.0013) and *fat‐5* suppression (0.54‐fold, *p* = 0.0017) (Figure 5R). The concomitant *clk‐1* upregulation (5.8‐fold, *p* = 0.0013), encoding a mitochondrial ubiquinone biosynthesis enzyme, suggests Medrysone enhances steroid‐mediated mitochondrial retrograde signaling while suppressing fatty acid desaturase (*fat‐5*) activity—a dual mechanism that may optimize membrane fluidity and redox homeostasis (Du et al. 2018). Notably, *spb‐1* (SPFH domain protein) downregulation (0.56‐fold, *p* = 0.0028) implies reduced lipid raft stabilization, potentially sensitizing cells to stress‐induced hormesis (Ana et al. 2024).

Polyphyllin VI's enrichment in response to organonitrogen compounds and *ucp‐4* overexpression (10.89‐fold, *p* < 0.0001) (Figure 5S) indicates mitochondrial uncoupling as a central mechanism. The *clk‐1* suppression (0.06‐fold, *p* = 0.0003) disrupts electron transport chain (ETC) efficiency, while *gst‐4* downregulation (0.47‐fold, *p* < 0.0001) attenuates glutathione conjugation capacity, collectively inducing a controlled metabolic crisis. This “pseudo‐hypoxic” state likely activates AMPK‐independent mitohormesis, where *ucp‐4*‐mediated proton leakage reduces ROS generation despite compromised ETC function, explaining its paradoxical lifespan extension without overt antioxidant induction (Cho et al. 2016).

Thymoquinone's association with synaptic transmission, cholinergic correlates with *unc‐29* (nicotinic acetylcholine receptor subunit) upregulation (2.13‐fold, *p* < 0.0001) and *dod‐17* overexpression (3.96‐fold, *p* = 0.007), a dopamine‐responsive GPCR (Figure 5T). *clk‐1* induction (11.63‐fold, *p* < 0.0001) synergizes with enhanced cholinergic signaling to elevate mitochondrial NADH oxidation capacity, creating a metabolic sink that buffers age‐related acetyl‐CoA accumulation—a known inhibitor of autophagy (Miyadera et al. 2002). This dual neuromodulatory‐mitochondrial axis likely underlies Thymoquinone's locomotor preservation (Figure 5C), as improved neuromuscular junction efficacy counteracts sarcopenia.

### 3.9 Limitations of ElixirSeeker

Despite the advantages of ElixirSeeker in lifespan‐extending compound screening, its design has several critical limitations that warrant consideration. First, the model's interpretability is constrained by inherent limitations of the Phenotype‐Driven Drug Discovery (PDD) approach. ElixirSeeker identifies compounds associated with lifespan extension through structural‐phenotype correlations but fails to explicitly elucidate the biological pathways or molecular targets involved, which may limit its application in mechanism‐based drug design or precision interventions. This limitation was previously mentioned.

Second, the study focuses solely on lifespan extension phenotypes without integrating multidimensional assessments like toxicity profiles or target specificity. This design choice was made because mature tools exist for predicting toxicity (e.g., toxicity classification, metabolic pathway simulation) and target interactions (e.g., molecular docking, network pharmacology), which can function as independent modules alongside ElixirSeeker. Since no existing models specifically address lifespan extension prediction well, this study prioritized filling that niche. Additionally, the absence of multi‐omics data (e.g., genomics, transcriptomics) may reduce the model's ability to capture complex aging mechanisms.

Compared to AgeXtend, a recently published model in *Nature Aging* (Arora et al. 2025), ElixirSeeker demonstrates distinct design trade‐offs. While AgeXtend focuses on predicting “broad anti‐aging activities” (e.g., reducing oxidative stress, improving metabolic homeostasis) by integrating multi‐omics data, ElixirSeeker strictly targets lifespan extension as the endpoint. This difference influences their performance: AgeXtend's broader scope may sometimes lead to discrepancies when applied to lifespan‐specific validation experiments. AgeXtend's multi‐omics integration enhances biological interpretability but comes with higher computational demands, making it less efficient for large‐scale compound screening.

In contrast, ElixirSeeker's lightweight ensemble learning framework allows rapid screening of tens of thousands of compounds on standard computing hardware, though it lacks mechanism‐based predictions. These differences create complementary roles: ElixirSeeker is good at identifying lifespan‐extending compounds through chemical feature analysis, while AgeXtend better supports pathway‐based research requiring mechanistic insights.

Regarding molecular fingerprint design, ElixirSeeker's approach effectively captures key patterns in small‐sample or sparse datasets, making it suitable for scenarios with limited data. This strategy not only benefits lifespan prediction but also offers a generalizable solution for other fields facing similar data challenges. However, its performance may decline when handling large datasets or complex biological mechanisms requiring multi‐omics integration.

Finally, both models depend on known aging mechanisms and may struggle with novel, uncharacterized pathways. For example, a compound extending lifespan through unknown epigenetic modifications (e.g., spatiotemporally specific histone acetylation changes) might not be predicted if such mechanisms are absent in training data.

Additionally, the machine learning model employed herein operates as a binary classifier, assigning scores based on the structural and physicochemical conformity of compounds to a dataset of known lifespan‐extending molecules. These scores reflect the likelihood of a compound's chemical features aligning with those of validated longevity agents, rather than directly predicting quantitative effects such as lifespan extension percentages.

## 4 Conclusion

In this study, we developed ElixirSeeker, a machine learning framework specifically engineered to address the challenges of small, sparse, and noisy biological datasets in lifespan‐extending compound discovery. Our model maximizes feature extraction from limited training data; this compact yet information‐rich representation captures critical structural and functional attributes of compounds, enabling predictions even under data scarcity. The framework's ability to disentangle latent pharmacological features from sparse inputs allowed the identification of four lifespan‐extending candidates—Medrysone, Polyphyllin VI, Thymoquinone, and Praeruptorin C—each with distinct mechanisms. This work establishes a way for machine learning to unlock hidden patterns in imperfect biological data, advancing virtual screening not only for lifespan‐extending compounds but also for broader applications characterized by sparse, noisy datasets.

## Author Contributions

Conceptualization, B.X. and Y.P.; methodology, Y.P. and H.C.; software, Y.P.; validation, F.Y., W.X., Z.H., J.Z., Y.G., and Y.L.; formal analysis, J.Z., Y.G., and Y.L.; investigation, F.Y., W.X. and S.L.; resources, B.X., F.Y., J.N., G.S. and J.Y.; data curation, Y.P.; writing – original draft preparation, Y.P., H.C. and F.Y.; writing – review and editing, F.Y., H.C., A.N.E., J.N. and H.L.; visualization, G.L., G.S.; supervision, J.Y. and B.X.; project administration, B.X. and J.N.; funding acquisition, B.X., F.Y., J.Y., J.N. and G.L. All authors have read and agreed to the published version of the manuscript.

## Ethics Statement

The authors have nothing to report.

## Consent

The authors have nothing to report.

## Conflicts of Interest

The authors declare no conflicts of interest.

## Supporting information


Appendix S1.

Appendix S2.

## Data Availability

The datasets analyzed during the current study are available via: https://github.com/Marissapy/ElixirSeeker‐ElixirFP.
