# Supplementary material for: ElixirSeeker: A Machine Learning Framework Utilizing Fusion Molecular Fingerprints for the Discovery of Lifespan‐Extending Compounds
Source: Aging Cell. 2025 May 26;24(8):e70116. doi: 10.1111/acel.70116 (PMC12341795; doi:10.1111/acel.70116)
Supplement: Supplementary file 1 — Appendix S1. [file ACEL-24-e70116-s002.pdf]

ElixirSeeker: A Machine Learning Framework Utilizing Fusion Molecular Fingerprints for the Discovery of Lifespan-Extending Compounds

Yan Pan[1,4,], Hongxia Cai[1,4,*], Fang Ye[1,3,*], Wentao Xu[4], Zhihang Huang[4], Jingyuan Zhu[4], Yiwen Gong[4], Yutong Li[4], Anastasia Ngozi Ezemaduka[4], Shan Gao[2], Shunqi Liu[5], Guojun Li[2,6], Hao Li[3], Jing Yang[4, ✉], Junyu Ning[2, ✉], Bo Xian[1,3,4, ✉]*

Appendix 1 Architecture Design

1. Background

The development of anti-aging drugs has long been constrained by the dual challenges of data scarcity and biological complexity. Although phenotypic drug discovery (PDD) is often criticized for its opaque mechanisms, its applicability in the field of anti-aging may be underestimated. Aging, as a systemic physiological decline, is inherently a "network disease" characterized by the dysregulation of multiple targets. The "black-box screening" approach of PDD may circumvent the limitations of traditional target-based drug discovery (TDD), which focuses on optimizing single targets. For example, known anti-aging compounds such as metformin and rapamycin exert their effects through multi-target interactions (e.g., AMPK-mTOR crosstalk). However, designing highly selective molecules for any single target via TDD may disrupt the robustness of the natural regulatory network. Therefore, PDD is naturally suited for anti-aging drug discovery.

Databases such as DrugAge systematically catalog validated active molecules, but their scale remains at the level of hundreds (in this study, the positive samples expanded from multiple sources only amount to 462), far below the tens of thousands of compounds typically required for conventional drug development. More critically, the multi-target regulatory nature of biological aging introduces significant noise into experimental data: differences in lifespan assessment systems across species (e.g., nematodes, mice), phenotypic lag effects, and experimental variability across platforms result in limited data contaminated with unvalidated potential active molecules and false positives.

At the molecular representation level, traditional fingerprint methods (e.g., Morgan, MACCS) can systematically encode structural features, but their high-dimensional sparse binary vectors sharply conflict with sparse samples. In high-dimensional space, redundant structural fragment noise can obscure critical pharmacophore signals, and fixed-length fingerprint designs further exacerbate the risk of information loss. For instance, conventional 1024-bit fingerprints in scenarios with hundreds of samples are highly prone to the curse of dimensionality, forcing models into the dilemma of overfitting or underfitting.

A deeper challenge arises from the multi-scale nature of anti-aging activity. A single molecule may delay aging through synergistic effects across multiple pathways, such as metabolic regulation, oxidative stress alleviation, and epigenetic modification. However, existing fingerprint systems struggle to dynamically capture such cross-level structure-function relationships. Additionally, biological differences in cross-species validation (e.g., target conservation between nematodes and mammals) limit the generalization ability of predictions based on single models.

In this context, the core innovation of ElixirSeeker lies in reconstructing the molecular representation paradigm—distilling anti-aging activity signals from high-dimensional noise through dynamic feature fusion and attention weight mechanisms. Its architecture

no longer relies on manually predefined fingerprint lengths or static structural fragments but instead employs machine learning-driven adaptive compression to retain key biologically relevant features while constructing a generalized representation system across species and targets. This balance between data efficiency and biological interpretability provides a new methodological foundation for anti-aging compound discovery in small-sample scenarios.

2. Molecular Fingerprints and XGBoost

One of the core innovations of ElixirSeeker lies in its molecular fingerprint fusion strategy. Molecular fingerprints are numerical representations of chemical structures, capable of transforming complex molecular information into machine-readable vectors. Different molecular fingerprints capture structural features of molecules from various perspectives, and thus, a single type of fingerprint often fails to comprehensively reflect the biological activity of molecules. To overcome this limitation, ElixirSeeker adopts a multi-fingerprint fusion strategy, combining the strengths of Morgan, Topological, and MACCS fingerprints to more comprehensively capture the structural information of molecules.

The Morgan fingerprint, also known as the circular fingerprint or Extended Connectivity Fingerprint (ECFP), is a molecular fingerprint based on atomic environments. It iteratively expands the surrounding atomic environment starting from each atom, generating a binary fingerprint that represents the presence or absence of specific structural fragments. The advantage of the Morgan fingerprint lies in its ability to capture local structural features of molecules, particularly excelling in identifying similar molecular fragments. Due to its iterative expansion nature, the Morgan fingerprint effectively captures the local chemical environment of molecules, making it highly effective in similarity searches and clustering analyses.

The Topological fingerprint, also known as the path fingerprint, is a molecular fingerprint based on topological paths in molecules. It represents topological paths or fragments in molecules, generating a binary fingerprint that captures both global and local structural features. Unlike the Morgan fingerprint, the Topological fingerprint emphasizes the global connectivity of molecules, effectively capturing the overall topological structure. Therefore, the Topological fingerprint excels in identifying structural similarities and pharmacophore features of molecules.

The MACCS fingerprint, short for Molecular ACCess System fingerprint, is a fixed-length molecular fingerprint based on predefined structural keys or pharmacophore patterns. It identifies the presence or absence of each predefined structural key, generating a binary fingerprint. The advantage of the MACCS fingerprint lies in its simplicity and information density, effectively capturing key pharmacophore features of molecules. Due to its predefined structural keys, the MACCS fingerprint excels in identifying known pharmacophores and structural patterns.

XGBoost (eXtreme Gradient Boosting) is an efficient gradient boosting framework widely used in machine learning and data science. Gradient boosting is an ensemble learning method that combines multiple weak learners (e.g., decision trees) into a strong learner. Its core idea is to iteratively optimize the predictive performance of the model by fitting the residuals of the previous step at each iteration, thereby gradually reducing prediction errors. XGBoost builds a powerful predictive model by integrating multiple weak learners (typically decision trees). The core idea of XGBoost is to iteratively add decision trees to optimize the model's performance, fitting the residuals of the previous step at each iteration to gradually reduce prediction errors. The advantages of XGBoost lie in its efficiency, flexibility, and scalability, enabling it to handle large-scale datasets and excel in various machine learning tasks.

The XGBoost algorithm is based on gradient boosting and regularization techniques. Gradient boosting optimizes the model's loss function by iteratively adding decision trees, fitting the residuals of the previous step at each iteration. Regularization prevents overfitting by adding penalty terms to the loss function, thereby improving the model's generalization ability. Additionally, XGBoost introduces several optimization techniques, such as parallel computing, sparse-aware algorithms, and weighted quantile sketches, further enhancing its computational efficiency and model performance.

3. Optimization of Molecular Fingerprint Length

The selection of molecular fingerprint length is not merely a technical detail; it directly affects the model's ability to represent molecular structures. Different fingerprint lengths influence the model's ability to capture molecular features. Shorter fingerprints may fail to comprehensively reflect the complex structures of molecules, especially when dealing with molecules with intricate topological structures or multiple pharmacophores, increasing the risk of information loss. On the other hand, longer fingerprints, while providing a more detailed description of molecular structures, may introduce redundant information, increase computational burden, and potentially lead to overfitting on training data, thereby reducing the model's generalization ability.

In the screening of anti-aging compounds, where datasets are typically small and noisy, selecting an appropriate fingerprint length is particularly important. Excessively long fingerprints may cause the model to lose focus in noise, failing to effectively identify true active compounds, while excessively short fingerprints may omit critical structural information, leading to inaccurate predictions of biological activity. Therefore, systematically determining the optimal length for each molecular fingerprint is a crucial step in ensuring the model's performance in anti-aging compound screening.

We employed the XGBoost algorithm to determine the optimal lengths for Morgan and Topological fingerprints. XGBoost is an efficient gradient boosting framework that optimizes model performance by iteratively adding decision trees. Its advantage lies not only in providing high-precision predictions but also in generating feature

importance scores, helping us understand the contribution of each feature to the model's predictions.

Specifically, we first performed a grid search on the lengths of Morgan and Topological fingerprints, ranging from 16 bits to 1016 bits with a step size of 8 bits. For each candidate length, we trained a model using the XGBoost algorithm and evaluated its performance through ten-fold cross-validation. The cross-validation results were assessed using the ROC AUC (Receiver Operating Characteristic Area Under the Curve) as the evaluation metric, which comprehensively reflects the model's classification performance, especially in handling imbalanced datasets.

The process of determining the optimal length is not only aimed at optimizing model performance but also provides a foundation for subsequent fingerprint fusion. By determining the optimal length for each fingerprint, we ensure that each fingerprint can contribute its unique structural information in the most effective way during the fusion process. For example, the 368-bit length of the Morgan fingerprint sufficiently captures local structural features, while the 696-bit length of the Topological fingerprint comprehensively reflects the global topological structure of molecules.

Moreover, the confirmation of optimal lengths ensures the stability of the model. By using cross-validation, we ensure that the selected lengths perform consistently across different data subsets, thereby reducing the model's dependency on specific datasets. This stability is particularly important in anti-aging compound screening, where datasets are typically small and noisy, and the model's stability directly affects its reliability in practical applications.

4. Fusion of Molecular Fingerprints

To combine the strengths of Morgan, Topological, and MACCS fingerprints, ElixirSeeker adopts two fusion strategies: PCA fusion and attention-driven KPCA fusion.

4.1.PCA Fusion

PCA (Principal Component Analysis) is a linear dimensionality reduction technique that projects high-dimensional data into a low-dimensional space while retaining the main features of the data. In ElixirSeeker, Morgan, Topological, and MACCS fingerprints are first concatenated to form a high-dimensional feature vector. Then, PCA is used to project these feature vectors into a low-dimensional space, generating a fused fingerprint called ElixirFP.

4.2.Attention-Driven KPCA Fusion

To further optimize feature representation, ElixirSeeker introduces an attention-driven KPCA (Kernel Principal Component Analysis) fusion strategy. KPCA is a nonlinear dimensionality reduction technique that projects data into a high-dimensional space through a kernel function, thereby capturing nonlinear relationships in the data. In

ElixirSeeker, KPCA employs a Gaussian Radial Basis Function (RBF) as the kernel function and incorporates feature importance scores generated by the XGBoost algorithm as weights. This strategy prioritizes fingerprint bits that contribute more to predicting anti-aging activity, generating a more compact and information-rich fused fingerprint called Attention-ElixirFP.

Algorithm 1 KPCA for Dimensionality Reduction of Molecular Fingerprints

Input: $\boldsymbol{X}_{Morgan}$, $\boldsymbol{X}_{Topological}$, $\boldsymbol{X}_{MACCS}$, γ, $\boldsymbol{n_{components}}$.

Output: *Feature vector after dimensionality reduction* $\boldsymbol{Reduced_{features}}$.

$\boldsymbol{X} = [\boldsymbol{X}_{Morgan}, \boldsymbol{X}_{Topological}, \boldsymbol{X}_{MACCS}]$

Calculate pairwise kernel matrix: $\boldsymbol{K}(\mathrm{i}, \mathrm{j}) = \exp\left(-\gamma|x_i - x_j|^2\right)$

Center the kernel matrix: $\boldsymbol{K} = \boldsymbol{K} - \frac{1}{n}\boldsymbol{1K} - \frac{1}{n}\boldsymbol{K1} + \frac{1}{n^2}\boldsymbol{1K1}$

Compute eigenvectors and eigenvalues: $\boldsymbol{Kv} = \boldsymbol{\lambda v}$

Select the top $\boldsymbol{n_{components}}$ eigenvectors.

$\boldsymbol{Reduced_{features}} = \boldsymbol{KV}$

Return: $\boldsymbol{Reduced_{features}}$.

5. Key Molecular Signals Determining Anti-Aging Properties

To explain why the PCA method enhances the model, as described earlier, we aim to find the important signals driving anti-aging activity of each molecular fingerprints. As shown in Figure A2.1A, Fingerprint information from lengths 1057 to 1100 is insignificant, indicating redundancy and noise. In contrast, fragments from 1101 - 1231, indicated by MACCS fingerprints, exhibit higher loading values, emphasizing their crucial role in indicating biological activity.

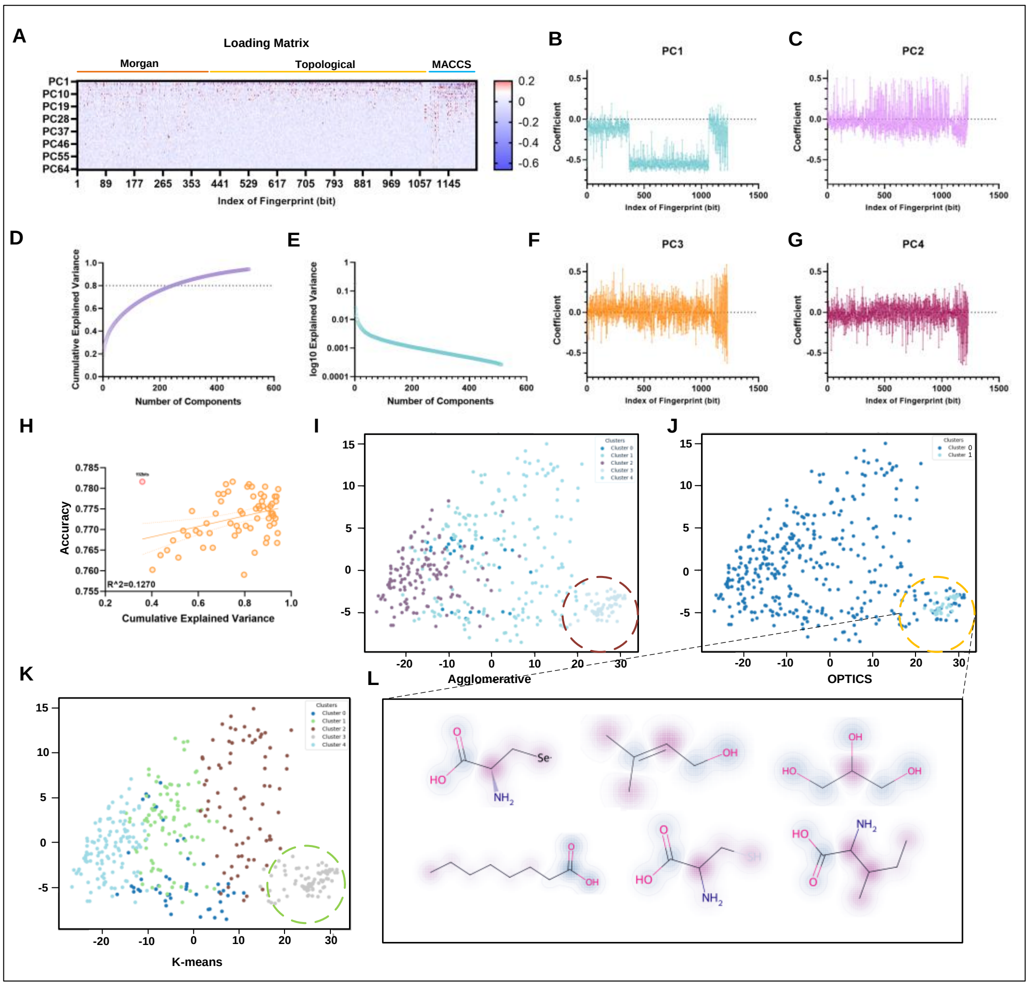

Figure A2.1: Detailed Analysis of PCA Components and Clustering of Molecular Fingerprints in Anti-Aging Compound Discovery. *(A) Loading matrix for the 64 components across Morgan, Topological, and MACCS fingerprints, highlighting the significant bits contributing to each component. (B-G) Coefficient plots for principal components PC1 (B), PC2 (C), PC3 (D), and PC4 (E), detailing the contribution of individual fingerprint bits to each principal component across the entire spectrum. (H) Scatter plot depicting the relationship between cumulative explained variance and model accuracy (I-K) KPCA-based clustering results, showing distinct clusters of molecular compounds colored by class: (I) Agglomerative clustering, (J) OPTICS clustering, and (K) K-means clustering. Each plot identifies nuanced grouping within the chemical space, with highlighted clusters indicating unique or significant features. (L) Representative molecular structures from key clusters identified in the OPTICS method.*

As shown in Figure A2.1A-C and F-G, the first principal component (PC1) has large and consistent negative coefficients, indicating common trends among features. Despite their diverse contributions, these features collectively highlight shared chemical properties. The coefficients distribution of the second to fourth principal components

is more uniform, with relatively smaller values, indicating balanced feature contributions and slight structural changes.

Figure A2.1D-E demonstrate that the first 64 principal components capture approximately 50% of the cumulative explained variation while maintaining good prediction accuracy. This indicates that these components effectively capture important anti-aging activity signals. Furthermore, we find essentially no correlation between cumulative explained variance and accuracy (Figure A2.1H). Despite having a relatively lower explained variance, ElixirFP generated by PCA significantly enhances model accuracy, suggesting that the anti-aging efficacy of small molecules is driven by key signals that are efficiently captured by these principal components.

This emphasizes the importance of using weighted KPCA for dimensionality reduction of molecular fingerprints, as it can reduce redundant features and noise, allowing the model to focus on important variables. Such an approach is valuable for screening bioactive small molecules in drug discovery, highlighting that key signals are often determined by a small subset of features.

6. Clustering Analysis of anti-aging compounds

As we delve deeper into understanding the landscape of anti-aging compounds, clustering analysis emerges as a powerful tool for unraveling intricate relationships among molecules. In this study, we characterized a series of anti-aging small molecules in the train set using 64-bit Attention-ElixirFP, to reveal subtle similarities and significant differences among these molecules.

We employed two distinctly different clustering algorithms: OPTICS, which represents unsupervised learning, and K-means and Agglomerative Clustering, which are commonly used in supervised learning. Consistent with previous studies, these methods show that anti-aging small molecules are difficult to categorize based on common characteristics. As shown in figures A2.1I-K, the OPTICS algorithm distinguished two significant groups of small molecules, but K-means and Agglomerative Clustering categorized the molecules into five classes on a finer scale. Different clustering methods for classifying anti-aging small molecules allow us to comprehend the interactions between molecules from different perspectives and infer possible biological properties based on their distribution in chemical space.

It is worth noting that, regardless of the clustering method used, molecules on the right side of the diagram are consistently classified as a separate group, suggesting a fundamental similarity in their chemical structures or biological functions. The tendency of these molecules to cluster in specific areas may indicate that they possess certain key biological activities or molecular properties.

In the aforementioned category (i.e., classified as Class 1 by OPTICS clustering), we observe that most molecules with anti-aging properties contain the carboxyl group (-

COOH), which prevalence may be related to its versatile biological functions in the body, as shown in table A2.1.

Compounds	SMILES
**Prenol**	CC(=CCO)C
**L-selenocysteine**	C([C@@H](C(=O)O)N)[Se]
**Glycine**	C(C(=O)O)N
**Valproic acid**	CCCC(CCC)C(=O)O
**L-serine**	C([C@@H](C(=O)O)N)O
**2-ketoglutaric acid**	C(CC(=O)O)C(=O)C(=O)O
**4-methyl-2-oxopentanoic acid**	CC(C)CC(=O)C(=O)O
**DL-alanine**	CC(N)C(=O)O
**3-methyl-2-oxopentanoic acid**	CCC(C)C(=O)C(=O)O
**3-methyl-2-oxobutanoic acid**	CC(C)C(=O)C(=O)O
**2-ketoglutaric acid**	O=C(O)CCC(=O)C(=O)O
**DL-Asparagine**	NC(=O)CC(N)C(=O)O
**β-alanine**	NCCC(=O)O
**Octanoic acid**	CCCCCCCC(=O)O
**Vaccenic acid**	CCCCCCC=CCCCCCCCCCC(=O)O
**DL-Cysteine**	NC(CS)C(=O)O
**3-hydroxybutyric acid**	CC(O)CC(=O)O
**2,2-dibromoacetic acid**	O=C(O)C(Br)Br
**2,2-dichloroacetic acid**	O=C(O)C(Cl)Cl
**Diethyl but-2-enedioate**	CCOC(=O)C=CC(=O)OCC
**Lactic acid**	CC(O)C(=O)O
**2-Butenedioic acid**	O=C(O)C=CC(=O)O
**Glycerol**	OCC(O)CO
**DL-Homocysteine**	NC(CCS)C(=O)O
**DL-Isoleucine**	CCC(C)C(N)C(=O)O
**DL-Leucine**	CC(C)CC(N)C(=O)O
**DL-Valine**	CC(C)C(N)C(=O)O
**DL-Lysine**	NCCCCC(N)C(=O)O
**DL-Methionine**	CSCCC(N)C(=O)O
**DL-Ornithine**	NCCCC(N)C(=O)O
**Oxalacetic acid**	O=C(O)CC(=O)C(=O)O
**9-hexadecenoice acid**	CCCCCCC=CCCCCCCCC(=O)O
**Pyruvic acid**	CC(=O)C(=O)O
**S-(Allylthio)-L-cysteine**	C=CCSSCC(N)C(=O)O
**Succinic acid**	O=C(O)CCC(=O)O
**DL-Threonine**	CC(O)C(N)C(=O)O
**2-hydroxypentanedioic acid**	O=C(O)CCC(O)C(=O)O

Table A2.1 Compounds from key clusters identified in the OPTICS method.

Carboxylic acids, as a critical component of cellular metabolism, play an important role in multiple key biochemical pathways, including but not limited to functioning as

metabolic intermediates, maintaining intracellular pH equilibrium, and participating in cell signaling. These characteristics make small molecules containing carboxyl groups ideal candidates for exploring anti-aging mechanisms. Therefore, the presence of the carboxyl group may be linked to their potential anti-aging effect, providing a valuable chemical foundation for the development of future anti-aging drugs.
