# Supplementary material for: ElixirSeeker: A Machine Learning Framework Utilizing Fusion Molecular Fingerprints for the Discovery of Lifespan‐Extending Compounds
Source: Aging Cell. 2025 May 26;24(8):e70116. doi: 10.1111/acel.70116 (PMC12341795; doi:10.1111/acel.70116)
Supplement: Supplementary file 2 — Appendix S2. [file ACEL-24-e70116-s001.pdf]

ElixirSeeker: A Machine Learning Framework Utilizing Fusion Molecular Fingerprints for the Discovery of Lifespan-Extending Compounds

Yan Pan[1,4,*] , Hongxia Cai[1,4,*], Fang Ye[1,3,*], Wentao Xu[4], Zhihang Huang[4], Jingyuan Zhu[4], Yiwen Gong[4], Yutong Li[4], Anastasia Ngozi Ezemaduka[4], Shan Gao[2], Shunqi Liu[5], Guojun Li[2,6], Hao Li[3], Jing Yang[4, ✉], Junyu Ning[2, ✉], Bo Xian[1,3,4, ✉]

Appendix 2 Supplementary Data

Catalogue

Catalogue 2

1 Supplementary Figures 3

2 Supplementary Tables 6

2.1. Candidates' Heatshock assay 6

2.2. Candidates' Lifespan assay 8

2.3. DMSO Survival data 10

2.4. Stability Analysis of Compounds 12

2.5. WormCNN-based lifespan prediction 12

2.6. Pharyngeal Pumping Assay of Candidates 13

2.7. Head Thrashing Assay of Candidates 15

2.8. Body Thrashing Assay of Candidates 17

2.9. Lipofuscin Assay of Candidates 19

3 Metadata 19

4 Predicted Targets of Candidates 33

5 Primers 35

6 TOP50 Candidates 38

1 Supplementary Figures

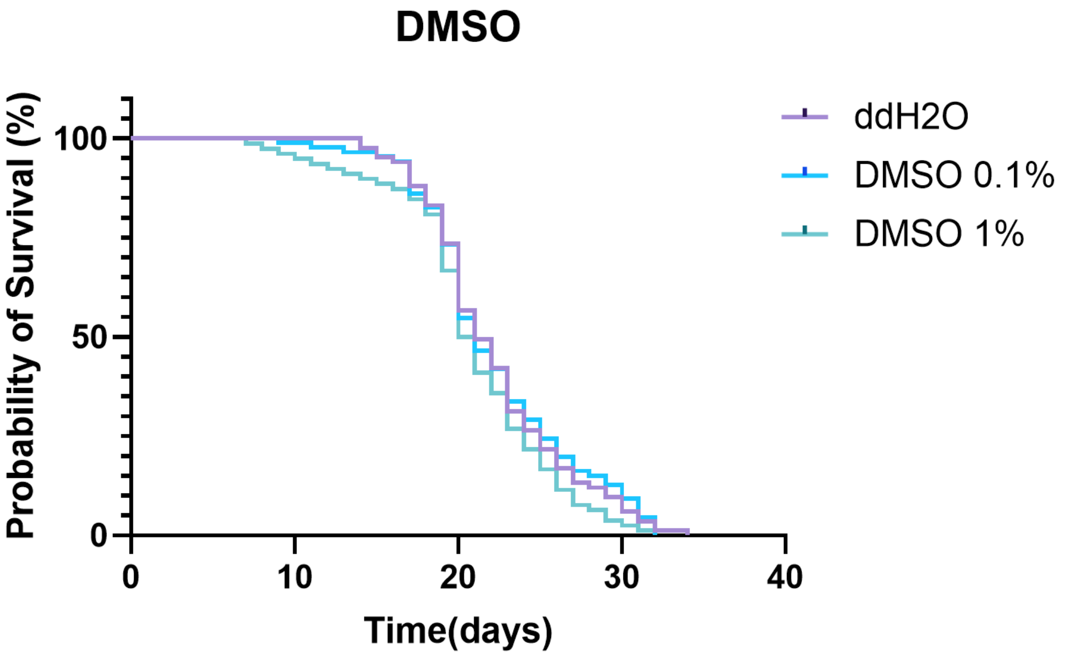

Figure S1 Kaplan-Meier lifespan curves for DMSO Treatment. *p-values calculated by log-rank test; Censored data (lost animals) are excluded from analysis.*

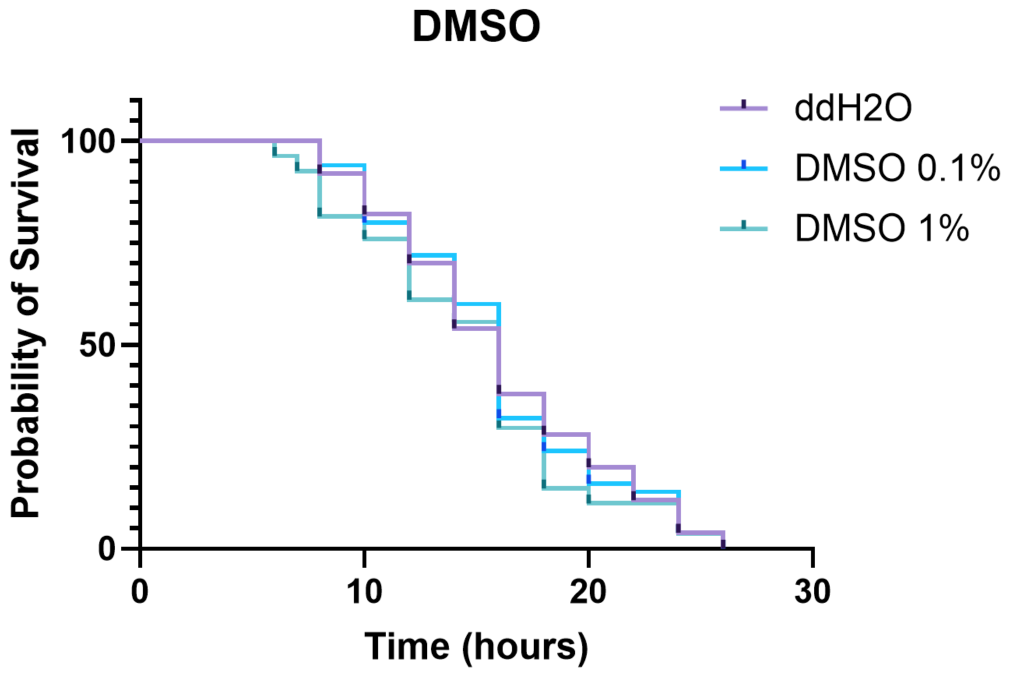

Figure S2 Kaplan-Meier lifespan curves for DMSO treatment under heatshock assay. *p-values calculated by log-rank test; Censored data (lost animals) are excluded from analysis.*

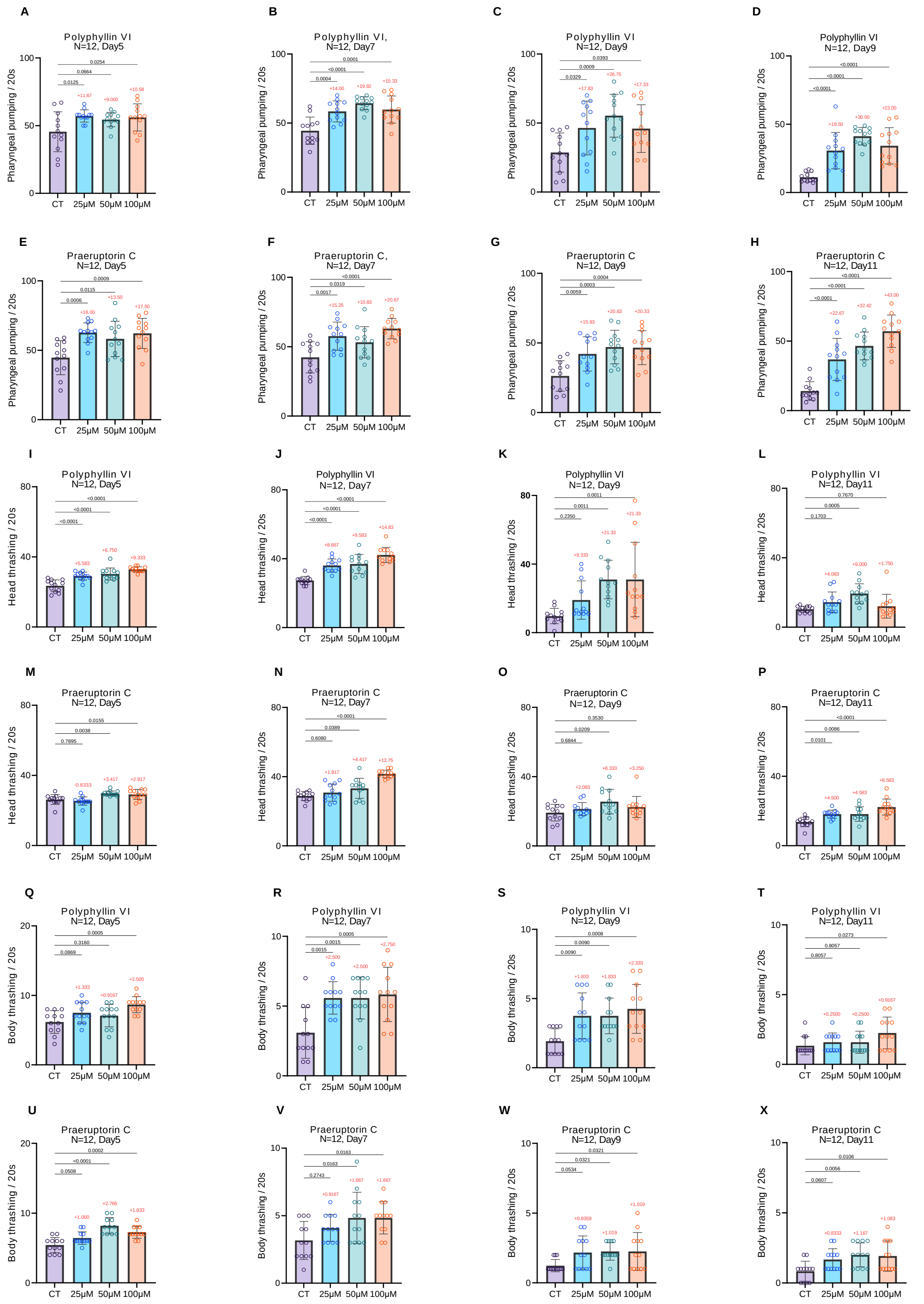

Figure S3. Quantitative assessments of pharyngeal pumping rate, body bending frequency, and head thrashing activity in control versus compound-treated group.

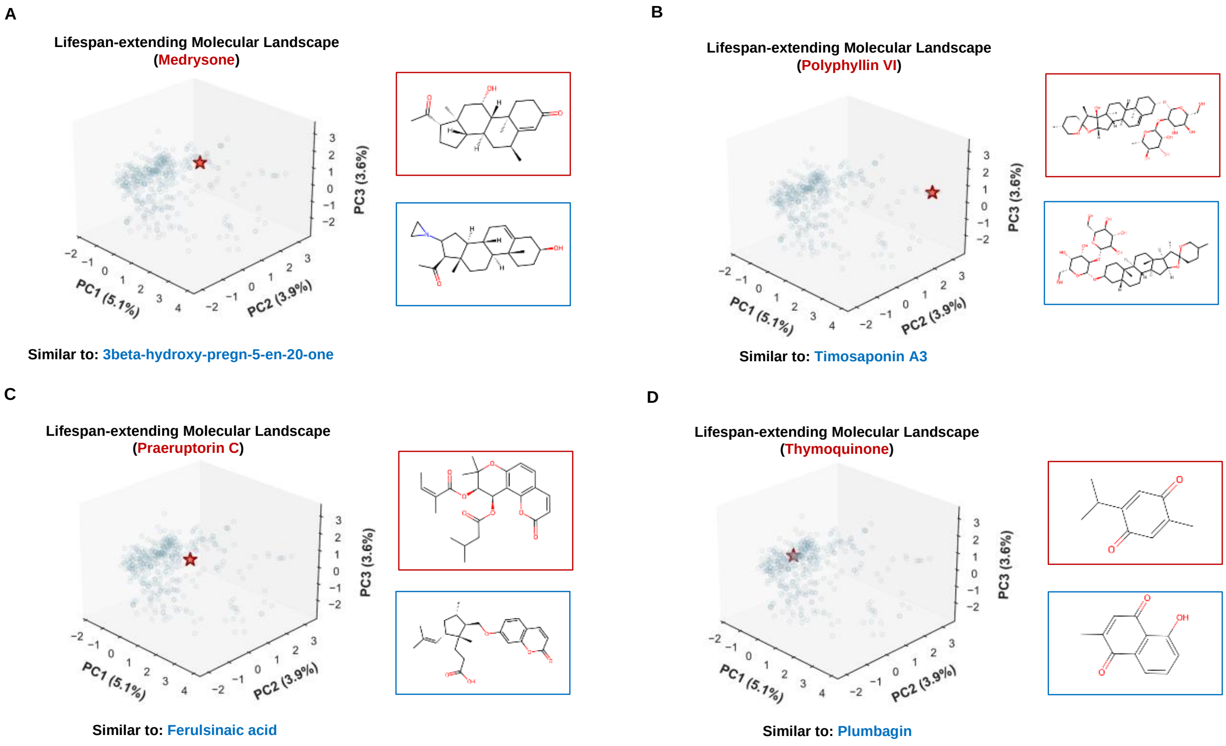

Figure S4. The PCA landscape of lifespan-extending compounds of DrugAge and the position of candidates.

2 Supplementary Tables

2.1. Candidates' Heatshock assay

Group	N	Mean Lifespan ± SD (hours)	Median Lifespan (hours)	Hazard Ratio (Logrank)	Log-rank p-value vs Control
Control	148	21.49±7.486	14	-	-
Polyphyllin VI 25μM	116	28.90±8.897	19	2.262	<0.0001****
Polyphyllin VI 50μM	90	26.62±6.622	16	1.528	<0.0001****
Polyphyllin VI 100μM	126	30.46±8.460	20	2.343	<0.0001****
Control	148	21.49±7.486	14	-	-
α-Hederin 25μM	120	22.75±6.750	16	1.146	0.1646 n.s.
α-Hederin 50μM	178	29.07±7.067	18	1.870	<0.0001****
α-Hederin 100μM	100	26.120±6.120	16	1.467	0.0002***
Control	148	21.49±7.486	14	-	-
7β-Hydroxylathyrol 25μM	117	21.72±7.718	16	1.112	0.2817 n.s.
7β-Hydroxylathyrol 50μM	101	27.64±7.644	18	1.792	<0.0001****
7β-Hydroxylathyrol 100μM	111	31.22±9.218	20	2.395	<0.0001****
Control	194	25.86±5.860	16	-	-
Thymoquinone 25μM	214	31.52±5.519	18	1.505	<0.0001****
Thymoquinone 50μM	175	31.51±5.509	18	1.501	0.0002***
Thymoquinone 100μM	240	30.88±6.881	18	1.605	0.0003***
Control	129	25.86±5.860	16		-
Medrysone 25μM	159	33.13±5.132	18	1.621	<0.0001****
Medrysone 50μM	200	29.20±5.200	16	1.288	0.0095**
Medrysone 100μM	157	31.37±5.369	18	1.501	<0.0001****
Control	129	25.86±5.860	16	-	-
Praeruptorin C 25μM	138	26.88±4.884	16	1.079	0.4697 n.s.

Group	N	Mean Lifespan ± SD (hours)	Median Lifespan (hours)	Hazard Ratio (Logrank)	Log-rank p-value vs Control
Praeruptorin C 50μM	186	35.32±7.323	21	2.070	<0.0001****
Praeruptorin C 100μM	144	37.29±9.278	24	2.343	<0.0001****

Table S1. The heatshock results and Kaplan-Meier (K-M) survival analysis of C. elegans exposed to varying concentrations of Polyphyllin VI, α-Hederin, 7β-Hydroxylathyrol, Thymoquinone and Medrysone (25 μM, 50 μM, and 100 μM) compared to the control group.

2.2. Candidates' Lifespan assay

Group	N	Mean Lifespan ± SD (days)	Median Lifespan (days)	Hazard Ratio (Logrank)	Log-rank p-value vs Control
Control	210	21.3 ± 3.4	20	-	-
Polyphyllin VI 25μM	222	25.8 ± 3.2	24	2.161	<0.0001****
Polyphyllin VI 50μM	189	25.2 ± 4.5	25	1.966	<0.0001****
Polyphyllin VI 100μM	186	23.8 ± 3.8	24	1.591	<0.0001****
Control	210	21.3±3.2	20	-	-
α-Hederin 25μM	195	19.0±4.5	18	0.732	0.0015**
α-Hederin 50μM	167	18.0±5.0	16	0.632	<0.001***
α-Hederin 100μM	185	20.3±3.5	20	0.831	0.144 n.s.
Control	210	21.3±3.2	20	-	-
7β-Hydroxylathyrol 25μM	207	20.9±3.3	20	0.906	0.064 n.s.
7β-Hydroxylathyrol 50μM	168	22.1±5.3	20	1.231	0.373 n.s.
7β-Hydroxylathyrol 100μM	186	21.6±4.9	21	1.234	0.919 n.s.
Control	194	15.4±4.1	20	-	-
Thymoquinone 25μM	214	17.5±3.5	22	1.413	<0.0005***
Thymoquinone 50μM	175	17.6±4.3	26	1.420	<0.0001****
Thymoquinone 100μM	240	17.0±3.4	26	1.197	<0.0001****
Control	184	24.2±6.7	20	-	-
Medrysone 25μM	186	24.9±5.0	26	0.9205	0.2602 n.s.
Medrysone 50μM	207	24.8±5.4	26	0.9622	0.3561 n.s.
Medrysone 100μM	201	27.6±6.2	28	1.507	<0.0001****
Control	210	21.5±3.1	20	-	-
Praeruptorin C 25μM	165	21.7±3.9	24	1.064	0.4504 n.s.
Praeruptorin C 50μM	219	24.2±4.0	24	2.508	<0.0001****

Group	N	Mean Lifespan ± SD (days)	Median Lifespan (days)	Hazard Ratio (Logrank)	Log-rank p-value vs Control
Praeruptorin C 100μM	183	22.3±3.5	27	1.225	0.0119*

Table S2. The heatshock results and Kaplan-Meier (K-M) survival analysis of C. elegans exposed to varying concentrations of Praeruptorin C, Polyphyllin VI, α-Hederin, 7β-Hydroxylathyrol, Thymoquinone and Medrysone (25 μM, 50 μM, and 100 μM) compared to the control group.

2.3. DMSO Survival data

Group	N	Mean Lifespan ± SD (days)	Median Lifespan (days)	Hazard Ratio (Logrank, A/D)	Log-rank p-value vs Control
Control(ddH2O)	259	22.3±4.0	20.0	-	-
DMSO 0.1%	256	23.1±4.3	21.0	1.040	0.9673 n.s.
DMSO 1%	264	22.1±3.6	20.5	0.8185	0.2241 n.s.

Table S3. The lifespan results and Kaplan-Meier (K-M) survival analysis of C. elegans exposed to varying concentrations of DMSO (1%, 0.1%) compared to the control group.

Time(days)	ddH2O	DMSO 0.1%	DMSO 1%
0	100.00	100.00	100.00
7			98.72
8			97.44
9		98.84	96.15
10			94.87
11		97.67	93.59
12			92.31
13		96.51	91.03
14	97.59		89.74
15	95.18	95.35	88.46
16	93.98	94.19	87.18
17	87.95	86.05	84.62
18	83.13	82.56	80.77
19	73.49	73.26	66.67
20	56.63	54.65	50.00
21	49.40	46.51	41.03
22	42.17	41.86	35.90
23	31.33	33.72	26.92
24	26.51	29.07	21.79
25	21.69	24.42	16.67
26	16.87	19.77	11.54
27	13.25	16.28	7.69
28	12.05	15.12	6.41
29	9.64	12.79	3.85
30	6.02	9.30	2.56
31	3.61	4.65	1.28
32	1.20	0.00	0.00
34	0.00		

Table S4. Survival Percentage of C. elegans Over Time Under Different Concentrations of DMSO

Group	N	Mean Lifespan ± SD (days)	Median Lifespan (days)	Hazard Ratio (Logrank, A/D)	Log-rank p-value vs Control
Control(ddH2O)	102	17.3±4.3	16	-	
DMSO 0.1%	91	17.1±4.2	16	1.002	0.9861 n.s.
DMSO 1%	108	15.8±4.8	16	0.8744	0.2901 n.s.

Table S5. The heatshock results and Kaplan-Meier (K-M) survival analysis of C. elegans exposed to varying concentrations of DMSO (1%, 0.1%) compared to the control group.

Time (hours)	ddH2O	DMSO 0.1%	DMSO 1%
0	100.00	100.00	100.00
6			96.30
7			92.59
8	92.00	94.00	81.48
10	82.00	80.00	75.93
12	70.00	72.00	61.11
14	54.00	60.00	55.56
16	38.00	32.00	29.63
18	28.00	24.00	14.81
20	20.00	16.00	11.11
22	12.00	14.00	
24	4.00	4.00	3.70
26	0.00	0.00	0.00

Table S6. Survival Percentage of C. elegans Over Time Under Different Concentrations of DMSO(heatshock).

2.4. Stability Analysis of Compounds

Compound Name	Original Rank (Full DB)	New Rank (Curated DB)	Rank Δ	Experiment Validated?
**Praeruptorin C**	1	6	-5	Yes
**Medrysone**	11	5	+6	Yes
**7β-Hydroxylathyrol**	13	44	-31	n.s.
**Thymoquinone**	14	32	-16	Yes
**α-Hederin**	12	19	-7	n.s.
**Polyphyllin VI**	5	4	+1	Yes

Table S7. Stability Analysis of Top-Ranking Compounds Across Database Versions.

2.5. WormCNN-based lifespan prediction

Treatment Group	Control (Vehicle)	Praeruptorin C (25µM)	Polyphyllin VI (50µM)	p-value vs Own Control
Predicted Lifespan vs Standard	13.2 ± 1.8	11.6 ± 1.1	10.9 ± 0.9	0.012
Δ	-	+39.3%	+24.1%	0.006

Table S8. WormCNN-based lifespan prediction at Day 11.

2.6. Pharyngeal Pumping Assay of Candidates

Group	Day	N	Mean Difference	95% CI of MD	p.adj	Significance
CT vs Praeruptorin C 25μM	5	12	-18.00	-28.86,-7.135	0.0006	Yes
CT vs Praeruptorin C 50μM	5	12	-13.50	-23.36,-2.635	0.0115	Yes
CT vs Praeruptorin C 100μM	5	12	-17.50	-28.36,-6.635	0.0009	Yes
CT vs Polyphyllin VI 25μM	5	12	-11.67	-21.15,-2.179	0.0125	Yes
CT vs Polyphyllin VI 50μM	5	12	-9.000	-18.49,0.4876	0.0664	No
CT vs Polyphyllin VI 100μM	5	12	-10.58	-20.07,-1.096	0.0254	Yes
CT vs Praeruptorin C 25μM	7	12	-15.25	-25.30,-5.199	0.0017	Yes
CT vs Praeruptorin C 50μM	7	12	-10.83	-20.88,-0.7827	0.0319	Yes
CT vs Praeruptorin C 100μM	7	12	-20.67	-30.72,-10.62	<0.0001	Yes
CT vs Polyphyllin VI 25μM	7	12	-14.00	-22.23,-5.770	0.0004	Yes
CT vs Polyphyllin VI 50μM	7	12	-19.92	-28.15,-11.69	<0.0001	Yes
CT vs Polyphyllin VI 100μM	7	12	-15.33	-23.56,-7.103	0.0001	Yes
CT vs Praeruptorin C 25μM	9	12	-15.83	-27.63,-4.032	0.0058	Yes
CT vs Praeruptorin C 50μM	9	12	-20.83	-32.63,-9.032	0.0003	Yes
CT vs Praeruptorin C 100μM	9	12	-20.33	-32.13,-8.532	0.0004	Yes
CT vs Polyphyllin VI 25μM	9	12	-17.83	-34.46,-1.203	0.0329	Yes
CT vs Polyphyllin VI 50μM	9	12	-26.75	-43.38,-10.12	0.0009	Yes
CT vs Polyphyllin VI 100μM	9	12	-17.33	-33.96,-0.7026	0.0393	Yes
CT vs Praeruptorin C 25μM	11	12	-22.67	-33.92,-11.42	<0.0001	Yes
CT vs Praeruptorin C 50μM	11	12	-32.42	-43.67,-21.17	<0.0001	Yes

Group	Day	N	Mean Difference	95% CI of MD	p.adj	Significance
CT vs Praeruptorin C 100μM	11	12	-43.00	-54.25,-31.75	<0.0001	Yes
CT vs Polyphyllin VI 25μM	11	12	-19.50	-29.53,-9.467	<0.0001	Yes
CT vs Polyphyllin VI 50μM	11	12	-30.00	-40.03,-19.97	<0.0001	Yes
CT vs Polyphyllin VI 100μM	11	12	-23.00	-33.03,-12.97	<0.0001	Yes

Table S9. Statistical comparison of Pharyngeal pumping effects under Praeruptorin C and Polyphyllin VI using Dunnett's post hoc test following one-way ANOVA.

2.7. Head Thrashing Assay of Candidates

Group	Day	N	Mean Difference	95% CI of MD	p.adj	Significance
CT vs Praeruptorin C 25μM	5	12	-0.8333	-1.627,3.294	0.7895	No
CT vs Praeruptorin C 50μM	5	12	-3.417	-5.877,-0.9559	0.0038	Yes
CT vs Praeruptorin C 100μM	5	12	-2.917	-5.377,-0.4559	0.0155	Yes
CT vs Polyphyllin VI 25μM	5	12	-5.583	-8.319,-2.847	<0.0001	Yes
CT vs Polyphyllin VI 50μM	5	12	-6.750	-9.486,-4.014	<0.0001	Yes
CT vs Polyphyllin VI 100μM	5	12	-9.333	-12.07,-6.597	<0.0001	Yes
CT vs Praeruptorin C 25μM	7	12	-1.917	-6.159,2.325	0.6080	No
CT vs Praeruptorin C 50μM	7	12	-4.417	-8.659,-0.1748	0.0389	Yes
CT vs Praeruptorin C 100μM	7	12	-12.75	-16.99,-8.508	<0.0001	Yes
CT vs Polyphyllin VI 25μM	7	12	-8.667	-12.84,-4.497	<0.0001	Yes
CT vs Polyphyllin VI 50μM	7	12	-9.583	-13.75,-5.414	<0.0001	Yes
CT vs Polyphyllin VI 100μM	7	12	-14.83	-19.00,-10.66	<0.0001	Yes
CT vs Praeruptorin C 25μM	9	12	-2.083	-7.603,3.436	0.6844	No
CT vs Praeruptorin C 50μM	9	12	-6.333	-11.85,-0.8136	0.0209	Yes
CT vs Praeruptorin C 100μM	9	12	-3.250	-8.770,-2.270	0.3530	No
CT vs Polyphyllin VI 25μM	9	12	-9.333	-22.87,-4.199	0.2350	No
CT vs Polyphyllin VI 50μM	9	12	-21.33	-34.87,-7.801	0.0011	Yes
CT vs Polyphyllin VI 100μM	9	12	-21.33	-34.87,-7.801	0.0011	Yes
CT vs Praeruptorin C 25μM	11	12	-4.500	-8.065,-0.9350	0.0101	Yes
CT vs Praeruptorin C 50μM	11	12	-4.583	-8.148,-1.018	0.0086	Yes

Group	Day	N	Mean Difference	95% CI of MD	p.adj	Significance
CT vs Praeruptorin C 100μM	11	12	-8.583	-12.15,-5.018	<0.0001	Yes
CT vs Polyphyllin VI 25μM	11	12	-4.083	-9.443,1.276	0.17.03	No
CT vs Polyphyllin VI 50μM	11	12	-9.000	-14.36,-3.640	0.0005	Yes
CT vs Polyphyllin VI 100μM	11	12	-1.750	-7.110,-3.610	0.7670	No

Table S10. Statistical comparison of Head thrashing effects under Praeruptorin C and Polyphyllin VI using Dunnett's post hoc test following one-way ANOVA.

2.8. Body Thrashing Assay of Candidates

Group	Day	N	Mean Difference	95% CI of MD	p.adj	Significance
CT vs Praeruptorin C 25µM	5	12	-1.000	-2.003,0.002971	0.0508	No
CT vs Praeruptorin C 50µM	5	12	-2.765	-3.791,-1.740	<0.0001	Yes
CT vs Praeruptorin C 100µM	5	12	-1.833	-2.836,-0.8304	0.0002	Yes
CT vs Polyphyllin VI 25µM	5	12	-1.333	-2.818,0.1509	0.0869	No
CT vs Polyphyllin VI 50µM	5	12	-0.9167	-2.401,0.5676	0.3160	No
CT vs Polyphyllin VI 100µM	5	12	-2.500	-3.984,-1.016	0.0005	Yes
CT vs Praeruptorin C 25µM	7	12	-0.9167	-2.321,0.4873	0.2743	No
CT vs Praeruptorin C 50µM	7	12	-1.667	-3.071,-0.2627	0.0163	Yes
CT vs Praeruptorin C 100µM	7	12	-1.667	-3.071,-0.2627	0.0163	Yes
CT vs Polyphyllin VI 25µM	7	12	-2.500	-4.129,-0.8705	0.0015	Yes
CT vs Polyphyllin VI 50µM	7	12	-2.500	-4.129,-0.8705	0.0015	Yes
CT vs Polyphyllin VI 100µM	7	12	-2.750	-4.379,-1.121	0.0005	Yes
CT vs Praeruptorin C 25µM	9	12	-0.9359	-1.883,0.01114	0.0534	No
CT vs Praeruptorin C 50µM	9	12	-1.019	-1.966,-0.07219	0.0321	Yes
CT vs Praeruptorin C 100µM	9	12	-1.019	-1.966,-0.07219	0.0321	Yes
CT vs Polyphyllin VI 25µM	9	12	-1.833	-3.267,-0.3997	0.0090	Yes
CT vs Polyphyllin VI 50µM	9	12	-1.833	-3.267,-0.3997	0.0090	Yes
CT vs Polyphyllin VI 100µM	9	12	-2.333	-3.767,-0.8997	0.0008	Yes
CT vs Praeruptorin C 25µM	11	12	-0.8333	-1.697,0.03010	0.0607	No
CT vs Praeruptorin C 50µM	11	12	-1.167	-2.030,-0.3032	0.0056	Yes

Group	Day	N	Mean Difference	95% CI of MD	p.adj	Significance
CT vs Praeruptorin C 100μM	11	12	-1.083	-1.947,-0.2199	0.0106	Yes
CT vs Polyphyllin VI 25μM	11	12	-0.2500	-1.080.0.5803	0.8057	No
CT vs Polyphyllin VI 50μM	11	12	-0.2500	-1.080,0.5803	0.8057	No
CT vs Polyphyllin VI 100μM	11	12	-0.9167	-1.747,-0.08632	0.0273	Yes

Table S11. Statistical comparison of Body thrashing effects under Praeruptorin C and Polyphyllin VI using Dunnett's post hoc test following one-way ANOVA.

2.9. Lipofuscin Assay of Candidates

Group	Day	N	Mean Difference	95% CI of MD	p.adj	Significance
CT vs Praeruptorin C 25μM	11	10	421.2	170.9,671.6	0.0006	Yes
CT vs Praeruptorin C 50μM	11	10	476.0	225.6,726.4	0.0001	Yes
CT vs Praeruptorin C 100μM	11	10	482.8	232.4,733.1	<0.0001	Yes
CT vs Polyphyllin VI 25μM	11	10	416.8	191.4,642.2	0.0002	Yes
CT vs Polyphyllin VI 50μM	11	10	370.4	145.0,595.9	0.0008	Yes
CT vs Polyphyllin VI 100μM	11	10	345.7	120.3,571.1	0.0017	Yes

Table S12. Statistical comparison of Lipofuscin effects under Praeruptorin C and Polyphyllin VI using Dunnett's post hoc test following one-way ANOVA.

3 Metadata

CT	Praeruptorin C 25μM	Praeruptorin C 50μM	Praeruptorin C 100μM
48	63	43	52
36	70	58	58
56	58	48	50
46	59	54	63
54	48	45	66
59	71	55	40
58	73	67	60
50	55	63	77
44	63	43	75
37	64	84	66
21	64	66	69
27	64	72	70

Table S13. The metadata of Pharyngeal pumping frequency (counts/20s) under Praeruptorin C (5days treatment)

CT	Praeruptorin C 25μM	Praeruptorin C 50μM	Praeruptorin C 100μM
28	63	62	68
54	65	57	60
32	59	54	72
40	64	42	52
58	48	37	68
52	44	48	63
50	70	54	62
37	47	56	54
47	59	45	59
33	54	77	77
25	74	42	64
52	44	64	57

Table S14. The metadata of Pharyngeal pumping frequency (counts/20s) under Praeruptorin C (7days treatment)

CT	Praeruptorin C 25μM	Praeruptorin C 50μM	Praeruptorin C 100μM
36	40	39	51
17	51	30	52
40	35	49	50
28	57	65	40
11	20	31	40
12	57	48	62
33	36	55	29
18	40	52	65
42	53	44	45
30	32	52	39
32	55	66	27
16	29	34	59

Table S15. The metadata of Pharyngeal pumping frequency (counts/20s) under Praeruptorin C (9days treatment)

CT	Praeruptorin C 25μM	Praeruptorin C 50μM	Praeruptorin C 100μM
11	44	67	55
13	64	33	64
23	41	36	47
13	24	54	57
30	47	41	62
17	56	42	35
12	39	58	70
6	28	50	59
8	40	38	43
9	22	44	52
11	24	42	64
16	12	53	77

Table S16. The metadata of Pharyngeal pumping frequency (counts/20s) under Praeruptorin C (11days treatment)

CT	Polyphyllin VI 25μM	Polyphyllin VI 50μM	Polyphyllin VI 100μM
59	58	57	39
46	56	54	43
54	58	53	61
66	66	62	64
67	63	49	56
43	50	58	45
50	50	42	54
33	56	53	57
40	57	53	56
42	58	60	57
25	58	55	69
21	56	58	72

Table S17. The metadata of Pharyngeal pumping frequency (counts/20s) under Polyphyllin VI (5days treatment)

CT	Polyphyllin VI 25μM	Polyphyllin VI 50μM	Polyphyllin VI 100μM
36	52	65	73
39	60	70	54
59	54	54	60
50	47	68	42
38	70	66	50
48	65	59	67
29	52	64	55
49	64	63	58
36	64	68	54
62	67	69	59
48	57	60	71
39	49	66	74

Table S18. The metadata of Pharyngeal pumping frequency (counts/20s) under Polyphyllin VI (7days treatment)

CT	Polyphyllin VI 25μM	Polyphyllin VI 50μM	Polyphyllin VI 100μM
25	65	53	42
35	50	53	23
8	20	80	36
14	15	38	23
28	62	63	35
40	70	28	70
7	56	70	35
47	29	40	72
45	27	66	68
45	39	72	43
22	66	46	57
27	58	55	47

Table S19. The metadata of Pharyngeal pumping frequency (counts/20s) under Polyphyllin VI (9days treatment)

CT	Polyphyllin VI 25μM	Polyphyllin VI 50μM	Polyphyllin VI 100μM
13	63	35	44
7	34	48	22
16	16	45	19
9	16	45	20
8	32	36	37
9	26	37	21
17	38	37	54
10	21	43	27
12	18	44	26
9	29	47	43
8	42	28	42
16	33	49	55

Table S20. The metadata of Pharyngeal pumping frequency (counts/20s) under Polyphyllin VI (11days treatment)

CT	Praeruptorin C 25μM	Praeruptorin C 50μM	Praeruptorin C 100μM
25	25	29	29
27	26	33	27
31	25	29	29
27	25	29	28
26	24	29	29
25	26	30	33
19	30	30	32
28	20	28	31
25	26	28	24
27	25	31	25
28	28	31	32
27	25	29	31

Table S21. The metadata of Head thrashing frequency (counts/20s) under Praeruptorin C (5days treatment)

CT	Praeruptorin C 25μM	Praeruptorin C 50μM	Praeruptorin C 100μM
23	30	45	43
26	32	36	41
30	36	35	45
27	25	38	41
32	38	34	40
31	28	25	45
32	38	25	39
30	26	26	38
27	35	32	40
29	28	35	42
30	24	33	43
29	29	35	42

Table S22. The metadata of Head thrashing frequency (counts/20s) under Praeruptorin C (7days treatment)

CT	Praeruptorin C 25μM	Praeruptorin C 50μM	Praeruptorin C 100μM
23	17	20	19
26	28	19	21
11	29	40	40
23	19	16	24
16	20	32	24
17	23	33	23
12	21	31	23
22	22	24	18
24	20	26	19
20	18	22	22
16	19	20	16
21	20	24	21

Table S23. The metadata of Head thrashing frequency (counts/20s) under Praeruptorin C (9days treatment)

CT	Praeruptorin C 25μM	Praeruptorin C 50μM	Praeruptorin C 100μM
16	19	21	20
7	18	26	24
15	14	21	16
18	19	16	28
14	20	24	33
12	23	11	21
16	19	15	22
14	15	17	22
12	17	19	20
13	18	16	24
14	19	16	19
13	17	17	18

Table S24. The metadata of Head thrashing frequency (counts/20s) under Praeruptorin C (11days treatment)

CT	Polyphyllin VI 25μM	Polyphyllin VI 50μM	Polyphyllin VI 100μM
27	30	30	33
26	30	31	32
27	28	29	33
25	29	27	32
19	29	32	30
20	27	26	32
25	32	32	32
24	28	29	34
28	29	39	32
21	24	32	35
22	31	28	35
18	32	28	34

Table S25. The metadata of Head thrashing frequency (counts/20s) under Polyphyllin VI (5days treatment)

CT	Polyphyllin VI 25μM	Polyphyllin VI 50μM	Polyphyllin VI 100μM
28	34	32	43
26	37	34	41
28	39	40	45
27	30	48	41
24	43	37	40
24	34	39	37
33	37	38	45
28	33	30	39
28	33	33	38
29	38	42	53
27	41	29	45
26	33	41	39

Table S26. The metadata of Head thrashing frequency (counts/20s) under Polyphyllin VI (7days treatment)

CT	Polyphyllin VI 25μM	Polyphyllin VI 50μM	Polyphyllin VI 100μM
10	11	18	21
12	37	16	14
10	11	21	21
18	12	27	12
7	11	29	25
1	40	24	9
8	14	31	23
6	32	38	52
8	11	44	33
9	24	42	64
11	13	53	77
16	12	29	21

Table S27. The metadata of Head thrashing frequency (counts/20s) under Polyphyllin VI (9days treatment)

CT	Polyphyllin VI 25μM	Polyphyllin VI 50μM	Polyphyllin VI 100μM
8	25	11	6
12	26	31	10
12	15	20	9
8	13	23	32
9	13	14	8
10	9	27	15
10	10	20	11
12	13	19	14
11	17	16	7
13	8	14	9
11	9	20	11
8	15	17	13

Table S28. The metadata of Head thrashing frequency (counts/20s) under Polyphyllin VI (11days treatment)

CT	Praeruptorin C 25μM	Praeruptorin C 50μM	Praeruptorin C 100μM
6	8	7	7
6	6	10	7
4	6	8	7
5	6	9	7
6	6	8	8
7	6	7	7
7	7	8	6
5	6	7	6
6	8	10	7
4	7	9	8
4	5	7	9
5	6		8

Table S29. The metadata of Body thrashing frequency (counts/20s) under Praeruptorin C (5days treatment)

CT	Praeruptorin C 25µM	Praeruptorin C 50µM	Praeruptorin C 100µM
2	4	6	3
1	4	7	6
2	5	3	5
5	5	9	4
3	6	4	5
4	3	5	5
2	3	3	4
4	4	5	7
5	5	4	3
3	3	3	5
2	3	6	6
5	4	3	5

Table S30. The metadata of Body thrashing frequency (counts/20s) under Praeruptorin C (7days treatment)

CT	Praeruptorin C 25µM	Praeruptorin C 50µM	Praeruptorin C 100µM
1	2	2	3
1	1	2	5
1	4	2	1
1	3	2	3
1	1	3	1
2	3	2	1
2	2	2	2
2	1	3	2
1	1	3	4
1	1	1	3
1	3	2	1
1	4	3	1
1			

Table S31. The metadata of Body thrashing frequency (counts/20s) under Praeruptorin C (9days treatment)

CT	Praeruptorin C 25μM	Praeruptorin C 50μM	Praeruptorin C 100μM
0	2	1	3
0	1	2	3
1	1	3	1
1	1	3	1
0	1	3	1
1	1	2	2
1	2	1	1
2	3	1	1
1	2	3	3
1	3	2	2
2	1	1	1
0	2	2	4

Table S32. The metadata of Body thrashing frequency (counts/20s) under Praeruptorin C (11days treatment)

CT	Polyphyllin VI 25μM	Polyphyllin VI 50μM	Polyphyllin VI 100μM
8	9	8	9
6	7	8	9
8	6	5	7
5	9	7	8
7	6	7	9
7	8	7	8
7	7	8	9
6	7	5	8
8	9	8	7
5	5	9	10
3	7	4	9
4	10	9	11

Table S33. The metadata of Body thrashing frequency (counts/20s) under Polyphyllin VI (5days treatment)

CT	Polyphyllin VI 25μM	Polyphyllin VI 50μM	Polyphyllin VI 100μM
2	5	2	5
2	6	5	3
5	5	6	6
1	7	6	6
7	6	7	6
4	4	5	8
2	5	4	9
5	6	7	4
2	4	5	8
3	6	7	5
3	8	6	3
1	5	7	7

Table S34. The metadata of Body thrashing frequency (counts/20s) under Polyphyllin VI (7days treatment)

CT	Polyphyllin VI 25μM	Polyphyllin VI 50μM	Polyphyllin VI 100μM
1	6	4	5
1	4	6	4
3	6	6	3
3	5	3	6
3	3	3	7
1	2	3	4
2	2	2	2
2	2	3	3
1	2	4	3
1	4	3	2
3	3	5	7
2	6	3	5

Table S35. The metadata of Body thrashing frequency (counts/20s) under Polyphyllin VI (9days treatment)

CT	Polyphyllin VI 25μM	Polyphyllin VI 50μM	Polyphyllin VI 100μM
2	3	2	3
2	1	3	2
1	2	2	3
1	1	1	4
1	2	1	3
1	2	1	1
1	1	1	1
1	1	1	1
3	2	2	2
1	1	1	4
1	1	3	1
1	2	1	2

Table S36. The metadata of Body thrashing frequency (counts/20s) under Polyphyllin VI (11days treatment)

CT	Praeruptorin C 25μM	Praeruptorin C 50μM	Praeruptorin C 100μM
2660.961	1632.524	1566.66	1174.268
2171.707	1955.784	1723.246	2152.918
2110.05	1841.719	2000.643	1925.64
2106.283	1799.863	2041.598	1810.227
2118.952	1681.871	1305.944	1541.202
1950.582	1700.253	1800.321	1650.123
2100.461	1850.713	1650.123	1700.456
1966.154	1810.192	1750.789	1750.789
2850.253	1800.582	1700.456	1800.321
2000.713	1750.461	1736.382	1702.366
2660.961	1632.524	1566.66	1174.268
2171.707	1955.784	1723.246	2152.918

Table S37. The metadata of Lipofuscin effects under Praeruptorin C (11days treatment)

CT	Polyphyllin VI 25μM	Polyphyllin VI 50μM	Polyphyllin VI 100μM
1319.55	1495.499	1340.769	1097.005
1327.88	1249.255	1438.716	1727.234
1472.57	1129.536	1316.187	1524.23
1757.07	1100.252	1312.163	1125.02
1447.01	1487.339	1236.179	1294.092
1500.12	1200.5	1250.4	1300.2
2000.46	1300.75	1300.75	1350.4
1800.79	1250.3	1350.2	1400.6
2200.32	1350.6	1400.6	1250.8
2166.26	1259.931	1341.864	1465.581
1319.55	1495.499	1340.769	1097.005
1327.88	1249.255	1438.716	1727.234

Table S38. The metadata of Lipofuscin effects under Polyphyllin VI (11days treatment)

4 Predicted Targets of Candidates

ADORA1	GRM5	MAPK10	PDE10A	CCNE2

Table S39. The predicted targets of Praeruptorin C.

PLK1	CHRM2	GSK3B	SLC6A2	MGLL
GLI2	CHRM1	PARP1	CA12	PTGS2
GLI1	CHRM3	CYP11B1	CTSK	TYMP
ALOX5	CA2	CYP11B2	TRPA1	AHR
CYP19A1	CA1	MCL1	ADH1B	PABPC1
PTPN2	ACHE	MAOA	ADH1C	SYK
MAOB	CHRNA7	MAP2K1	CTSS	P2RX7
CHRNA4	SHBG	DUSP1	CTSB	CNR1
CHRM4	SOAT1	HDAC8	SLC6A4	CASP1
CHRM5	ACE	ADH1A	RGS4	CYP2A6

Table S40. The predicted targets of Thymoquinone.

IL2	HSD11B1	CDK1	DGAT1	MERTK
STAT3	ADORA1	GLI1	MDH1	DRD4
PTAFR	TYMS	ADORA2A	MDH2	JUN
PTPN1	RORC	ALOX5AP	MMP2	HDAC6
PPM1B	SLC5A2	TTL	ADORA3	ADRB2
PPP1CC	SLC5A1	PFKFB3	ADRB1	DUSP3

PPP2CA	HSP90AA1	MET	TNF	ITGA2
PPP2R5A	S1PR3	HRH1	MMP13	ADAMTS4
BCL2L1	S1PR1	KCNH2	AXL	CDC25A
HSD11B2	PSEN2	CCR3	TYRO3	CDC25B

Table S41. The predicted targets of Polyphyllin Vl.

HSD11B2	SLC6A3	BACE1	ESR2	PTPN6
HSD11B1	ADORA3	FNTA	FABP1	BCHE
NR3C2	MAPK3	PTPN2	IL6	ESR1
CYP19A1	SIGMAR1	ALOX5	GLUL	HMGCR
SERPINA6	NPC1L1	NOS2	PTPN1	SRD5A2
SHBG	PTGES	SLC6A4	NR1I2	FDFT1
NR3C1	CDC25A	RORA	PTPRF	CD81
AR	PRKCH	POLB	PLA2G1B	NR1I3
PGR	PTPN11	HSD17B3	ACP1	RORC
CYP17A1	AKR1B10	IDO1	ATP12A	PDE4D

Table S42. The predicted targets of Medrysone

5 Primers

Name	Sequence	Function
sir2.1	CACGTGATAGTGGCAACGATTC	Epigenetic and transcriptional regulation.
pha-1	TTGCTGTCTCGAATGACCATCA	Epigenetic and transcriptional regulation.
elt-2	TCTGTACGACCCCAGTATTCCT	Epigenetic and transcriptional regulation.
bec-1	CCGTTGAGGTTGGATTTCACAC	Protein homeostasis and autophagy.
atg-101	GCTGGATATTTGCGAGCACATT	Protein homeostasis and autophagy.
rsks-1	TATCGAATACATGGCACCGGAG	Protein homeostasis and autophagy.
actin	TCGGTATGGGACAGAAGGAC	Housekeeping gene.
tubulin	TCAACACTGCCATCGCCGCC	Housekeeping gene.
clk-1	GCACATACTGCTGCTTCTCG	Mitochondrial function.
ifp-1	GATGTTCCAGCTGAGTTCTCCA	Mitochondrial function.
mev-1	CAATCCAGAAGTTCGGATGGGA	Mitochondrial function.
ucp-4	GCACCAGCAATCACAAGACATT	Mitochondrial function.
isp-1	ACACCAGCCGCCGATAAT	Mitochondrial function.
daf-16	ATCGGTGCCTTGTTCTTCATCT	Insulin signaling pathway.
daf-2	AAAAGATTTGGCTGGTCAGAGA	It belongs to the insulin-like receptor family and regulates lifespan, metabolism, and stress responses. It influences the activity of the DAF-16/FOXO transcription factor through the PI3K-Akt signaling pathway.
daf-18	TCGACACCGGAGAGCAAAAATA	Insulin signaling pathway.
age-1	AACACCTCTCTCTCCTCTCTCC	Insulin signaling pathway.
eat-2	AAAGGATTTGCGTGAGGGGTAT	Dietary restriction.
sod-3	GGCTAAGGATGGTGGAGAAC	Stress response.
skn-1	CACGCCGTCAGCGAAGTA	It regulates antioxidant and detoxification genes and is involved in the response to nitrogen compound metabolic stress.
hsp12.2	ACACCAAGGAAAAGTTCGAGGT	Stress response.
hif-1	GGTTTAGCACCGGAATTCGATG	Stress response.
cat-1	GGTTTAGCACCGGAATTCGATG	Neural transmission.
nhr-8	GACTCACCGCATACGAACC	Metabolism.
sek-1	ATGCTCGGTGAGTATTGG	Immunity.
dod-17	ACACGGACACGCATTACCA	Development.
ctl-2	ACACGGACACGCATTACCA	Stress response.
sek-1	GCCGATGGAAAGTGGTTTTA	Immunity.

Name	Sequence	Function
pmk-1	CCGACTCCACGAGAAGGATA	Stress response.
fipr-22	CCCAATCCAGTATGAAGTTG	Protein homeostasis and autophagy.
cnc-4	ATGCTTCGCTACATTCTCGT	Stress response.
daf-12	ATTCGATTCCCTTTCTGCCACT	Encoding the nuclear receptor DAF-12, which regulates fat metabolism and development through the dafachronic acid signaling pathway.
nhr-1	CACAAGTTGAAGGGCTGTGC	Encoding the nuclear receptor NHR-1, which is involved in regulating lipid metabolism and proteostasis.
sbp-1	TTTGGCTCTTCCGAACCTACTC	Homologs of the Sterol Regulatory Element-Binding Protein (SREBP) family regulate fatty acid synthesis and desaturation.
fat-5	GGATGGTTGTTGGTGAAGAAGC	Encoding Δ-9 fatty acid desaturase, which is involved in fatty acid synthesis.
acs-2	TGTAATGAGCCAAGTGCAGGAT	Key enzymes involved in fatty acid β-oxidation, regulating fat accumulation.
lipl-4	TGTTAACTATGTGGCCCAGCAT	Lipase, involved in lipid degradation and regulation of autophagy.
eat-4	CCTTCAAATCCTGAGGTTCGGA	Encoding glutamate transporters, involved in nitrogen metabolism-related neurosignaling.
hlh-30	CCTTCAAATCCTGAGGTTCGGA	Regulates lysosome function, involved in nitrogen metabolism waste clearance.
nhx-2	GGGCCATCAGTTTGACTGGA	Encoding the sodium/hydrogen exchanger, involved in ammonia excretion and acid-base balance regulation.
gst-4	GGCAAGAAAATTTGGACTCGCT	Glutathione S-transferase (GST) responds to oxidative stress generated by nitrogen metabolism.
let-23	TGATGCCAGTGTTCGACCAA	The epidermal growth factor receptor (EGFR) is encoded by a gene that regulates cell fate determination during development, such as the development of the vulva. Its signaling is transmitted through the downstream Ras-MAPK pathway.
kin-15	GGGGGAAGCGATGGTATTTTTG	The function of this protein is not yet fully understood. However, studies suggest that it may be involved in cell

Name	Sequence	Function
		migration and developmental regulation, and is related to the adaptive regulation of transmembrane receptor signaling pathways.
egl-15	CGTTGGTGATGAATCGACGC	The gene encoding fibroblast growth factor receptor (FGFR) is involved in cell migration and the development of the reproductive system. Its signaling is transmitted through the Ras-MAPK pathway and involves interactions with the extracellular matrix.
sem-5	TGCTTATCACAGAACCGCTTCT	As an adaptor protein, it connects the activated receptor tyrosine kinase (such as LET-23) with downstream signaling molecules (such as SOS-1), thereby promoting the activation of the Ras signaling pathway.
sos-1	TGCTTATCACAGAACCGCTTCT	It is a Ras guanine nucleotide exchange factor (GEF) that directly participates in the activation of the Ras-MAPK pathway in response to signaling from receptor tyrosine kinases.
unc-17	TTGCTATGGTTGGGTTGGCTAT	The vesicular acetylcholine transporter (VAChT) is responsible for the presynaptic release of acetylcholine.
cha-1	TTGCTATGGTTGGGTTGGCTAT	Choline acetyltransferase (ChAT) catalyzes the synthesis of acetylcholine.
unc-38	TGAGGATTGGAAATACGTGGCA	The α subunit of the nicotinic acetylcholine receptor (nAChR) mediates postsynaptic signal transduction.
unc-29	TGGCATTCTTGAGTATGGCTGT	The non-α subunit of the nicotinic acetylcholine receptor (nAChR) forms a functional receptor in conjunction with UNC-38.
ace-2	CCACTTCTGTGTGAATGCAAGG	Acetylcholinesterase (AChE) degrades acetylcholine in the synaptic cleft to terminate signaling.
ric-3	AATGGGATTCTTGGGTCGTTCA	The acetylcholine receptor chaperone protein promotes the proper assembly of receptor subunits.

Table S43 All primers used in this experiment and their functions

6 TOP50 Candidates

Score	Name	Description
0.716523	Praeruptorin C(this work)	Praeruptorin C is a main bioactive constituent of Peucedanum praeruptorum (also known as Bai-Hua Qian Hu). Praeruptorin C is a calcium antagonist.
0.708575	20-Deoxyingenol	20-Deoxyingenol is a bioactive diterpenoid with anticancer and anti-inflammatory properties, potentially acting as an inhibitor of protein kinase C (PKC) and other signaling pathways.
0.708465	Ginkgolid B(positive)	Ginkgolide B is a natural terpenoid compound that acts as a positive modulator of platelet-activating factor (PAF) receptor antagonism, exhibiting anti-inflammatory and neuroprotective effects.
0.707907	Praeruptorin E	Praeruptorin E is a natural coumarin compound that exhibits anti-inflammatory and vasodilatory effects, potentially acting as a calcium channel blocker.
0.705013	Polyphyllin VI(this work)	Polyphyllin VI is a steroidal saponin with anticancer and anti-inflammatory properties, acting as an inhibitor of key signaling pathways such as PI3K/AKT and NF-κB.
0.704307	Etoposide	Etoposide is a topoisomerase II inhibitor that induces DNA breakage and apoptosis, primarily used as a chemotherapeutic agent in cancer treatment.
0.703291	Triptolide(positive)	Triptolide is a natural diterpenoid triepoxide that acts as a positive modulator of cellular stress responses, exhibiting potent anti-inflammatory, immunosuppressive, and anticancer properties by inhibiting NF-κB and other key signaling pathways.
0.701729	Ginkgolid A	Ginkgolide A is a natural terpenoid compound that acts as a platelet-activating factor (PAF) receptor antagonist, exhibiting anti-inflammatory and neuroprotective effects.
0.701113	NAD+(positive)	NAD+ is a crucial coenzyme that acts as a positive regulator of sirtuins and other enzymes involved in cellular metabolism, DNA repair, and antioxidant defense.
0.700947	Pregnenolone(positive)	Pregnenolone is a neuroactive steroid that acts as a positive allosteric modulator of GABA_A receptors and exhibits neuroprotective and anti-inflammatory properties.

Score	Name	Description
0.699309	Medrysone(this work)	Medrysone is a synthetic corticosteroid with anti-inflammatory and immunosuppressive effects, acting as an agonist of glucocorticoid receptors to inhibit pro-inflammatory mediators.
0.69821	Alpha-Hederin(this work)	Alpha-Hederin is a triterpenoid saponin with anticancer and anti-inflammatory properties, acting as an inhibitor of key signaling pathways such as PI3K/AKT and NF-κB.
0.697395	Lathyrol	Lathyrol is a bioactive diterpenoid with anti-inflammatory and anticancer properties, potentially acting as an inhibitor of key signaling pathways such as NF-κB.
0.696004	7-beta-Hydroxylathyrol(this work)	7-beta-Hydroxylathyrol is a bioactive diterpenoid that exhibits anti-inflammatory and anticancer effects, potentially acting as an inhibitor of key signaling pathways such as NF-κB.
0.6956	Thymoquinone(this work)	Thymoquinone is a bioactive compound with antioxidant, anti-inflammatory, and anticancer properties, acting primarily as an inhibitor of NF-κB and other pro-inflammatory signaling pathways.
0.691123	Praeruptorin A	Praeruptorin A is a natural coumarin compound that exhibits anti-inflammatory and vasodilatory effects, potentially acting as a calcium channel blocker.
0.690073	Desonide	Desonide is a low-potency topical corticosteroid that acts as an anti-inflammatory agent by inhibiting the release of pro-inflammatory mediators and suppressing immune responses.
0.688038	Sauchinone(positive)	Sauchinone is a lignan compound that acts as a positive modulator of AMP-activated protein kinase (AMPK) and exhibits antioxidant and anti-inflammatory properties.
0.683224	Praeruptorin D	Praeruptorin D is a natural coumarin compound that exhibits anti-inflammatory and antioxidant effects, potentially acting as an inhibitor of NF-κB signaling pathways.
0.681651	Pteryxin	Pteryxin is a natural coumarin derivative known for its anti-inflammatory and antioxidant activities, potentially acting as an inhibitor of pro-inflammatory mediators.
0.67355	Griseofulvin	Griseofulvin is a antifungal agent that inhibits fungal cell mitosis by disrupting microtubule function.
0.673124	Brusatol	Brusatol is a natural compound that acts as an Nrf2 inhibitor, exhibiting potent antioxidant and anti-inflammatory properties.

Score	Name	Description
0.671984	Loteprednol etabonate	Loteprednol etabonate is a glucocorticoid receptor agonist with anti-inflammatory properties, designed to be rapidly metabolized to inactive metabolites after exerting its local effects, thereby reducing systemic side effects.
0.666266	Lindenenol	Lindenenol is a natural sesquiterpenoid that exhibits anti-inflammatory and antioxidant properties, primarily by inhibiting NF-κB and MAPK signaling pathways and scavenging free radicals to reduce oxidative stress and modulate inflammatory responses.
0.666144	Rapamycin(positive)	Rapamycin is a specific mTOR (mechanistic target of rapamycin) inhibitor that exerts immunosuppressive, anticancer, and anti-aging effects by blocking mTOR signaling, while also demonstrating indirect antioxidant properties through modulation of cellular metabolism and reduction of oxidative stress.
0.665706	Enoxolone	Enoxolone, also known as glycyrrhetinic acid, is a natural triterpenoid that functions as an anti-inflammatory and antioxidant agent, primarily by inhibiting NF-κB and MAPK signaling pathways and enhancing cellular antioxidant defenses through the upregulation of enzymes like superoxide dismutase (SOD).
0.665706	18alpha-Glycyrrhetinic acid(positive)	18alpha-Glycyrrhetinic acid is a natural triterpenoid that acts as an anti-inflammatory and antioxidant agent, primarily by inhibiting NF-κB and MAPK signaling pathways and enhancing cellular antioxidant defenses through the upregulation of enzymes like superoxide dismutase (SOD).
0.664748	5-O-Methylvisammioside	5-O-Methylvisammioside is a natural coumarin glycoside that exhibits anti-inflammatory and antioxidant properties, primarily by inhibiting NF-κB signaling and scavenging free radicals to reduce oxidative stress and modulate inflammatory responses.
0.663636	ingenol	Ingenol is a natural diterpene ester that acts as a protein kinase C (PKC) activator, exerting immunomodulatory and anticancer effects, while also demonstrating anti-inflammatory properties through modulation of signaling pathways such as NF-κB and MAPK.
0.6624	Cucurbitacin B(positive)	Cucurbitacin B is a natural triterpenoid that functions as a potent inhibitor of JAK/STAT and MAPK signaling pathways, exerting anticancer and anti-inflammatory effects, while also demonstrating antioxidant activity by reducing oxidative stress and modulating cellular redox balance.

Score	Name	Description
0.659802	Peoniflorin(positive)	Peoniflorin is a natural monoterpene glycoside that acts as an anti-inflammatory and neuroprotective agent, primarily by inhibiting NF-κB and MAPK signaling pathways, while also demonstrating antioxidant activity through the reduction of reactive oxygen species (ROS) and modulation of cellular redox balance.
0.659602	sec-o-Glucosylhamaudol	Sec-o-Glucosylhamaudol is a natural coumarin glycoside that exhibits anti-inflammatory and antioxidant properties, primarily by inhibiting NF-κB and MAPK signaling pathways and scavenging free radicals to reduce oxidative stress and modulate inflammatory responses.
0.658966	MLS006011132	MLS006011132 is a small-molecule inhibitor that targets specific signaling pathways, such as PI3K/AKT or MAPK, to exert anticancer and anti-inflammatory effects, while also demonstrating potential antioxidant activity through modulation of cellular redox balance.
0.657781	Hydroxysafflor Yellow A	Hydroxysafflor Yellow A is a natural flavonoid that acts as an anti-inflammatory and antioxidant agent, primarily by inhibiting NF-κB and MAPK signaling pathways and scavenging free radicals to reduce oxidative stress and protect against cellular damage.
0.656911	Hastatoside	Hastatoside is a natural iridoid glycoside that exhibits anti-inflammatory and antioxidant properties, primarily by inhibiting NF-κB signaling and scavenging free radicals to reduce oxidative stress and modulate inflammatory responses.
0.655584	teniposide	Teniposide is a topoisomerase II inhibitor that exerts its anticancer effects by stabilizing the DNA-topoisomerase II complex, leading to DNA strand breaks and apoptosis, without direct antioxidant or anti-inflammatory properties.
0.65469	ISODONOL	Isodonol is a natural diterpenoid that functions as an anti-inflammatory and anticancer agent, primarily by inhibiting NF-κB and MAPK signaling pathways, while also exhibiting antioxidant activity through the scavenging of free radicals and reduction of oxidative stress.
0.654011	hypocrellin A	Hypocrellin A is a natural photosensitizer that acts as a potent generator of reactive oxygen species (ROS) under light irradiation, exerting photodynamic anticancer and antimicrobial effects, while also demonstrating antioxidant properties in dark conditions by scavenging free radicals.

Score	Name	Description
0.653242	Metformin(positive)	Metformin is an AMP-activated protein kinase (AMPK) activator that primarily inhibits hepatic gluconeogenesis and improves insulin sensitivity, while also exerting antioxidant and anti-inflammatory effects through modulation of mitochondrial function and reduction of reactive oxygen species (ROS).
0.65221	Picroside II	Picroside II is a natural iridoid glycoside that acts as an anti-inflammatory and antioxidant agent, primarily by inhibiting NF-κB and MAPK signaling pathways and scavenging free radicals to reduce oxidative stress and protect against cellular damage.
0.652155	fusidic acid	Fusidic acid is a bacteriostatic antibiotic that functions as an inhibitor of bacterial protein synthesis by binding to elongation factor G (EF-G), preventing ribosomal translocation and thereby inhibiting bacterial growth.
0.6507	PHY34	PHY34 is a small-molecule inhibitor that targets the Wnt/β-catenin signaling pathway, exerting anticancer effects by suppressing β-catenin activity and downstream oncogenic gene expression, while also demonstrating potential antioxidant properties through modulation of cellular redox balance.
0.649231	tofogliflozin hydrate	Tofogliflozin hydrate is a selective sodium-glucose cotransporter 2 (SGLT2) inhibitor that reduces blood glucose levels by blocking glucose reabsorption in the kidneys, while also demonstrating potential cardioprotective and antioxidant effects through indirect mechanisms such as improved metabolic regulation and reduced oxidative stress.
0.646564	Madecassic Acid	Madecassic acid is a triterpenoid compound that functions as an anti-inflammatory and antioxidant agent, primarily by inhibiting NF-κB and MAPK signaling pathways and enhancing cellular antioxidant defenses through the upregulation of enzymes like superoxide dismutase (SOD) and glutathione peroxidase (GPx).
0.643038	Triptonide(positive)	Triptonide is a natural diterpenoid epoxide that acts as a potent inhibitor of NF-κB and STAT3 signaling pathways, exerting anti-inflammatory, anticancer, and immunomodulatory effects, while also demonstrating antioxidant activity by reducing oxidative stress and modulating cellular redox balance.
0.642729	Dehydroandrographolide succinate	Dehydroandrographolide succinate is a derivative of andrographolide that functions as an anti-inflammatory and immunomodulatory agent, primarily by inhibiting NF-κB and MAPK signaling pathways, while also demonstrating antioxidant activity through the reduction of reactive oxygen species (ROS) and enhancement of cellular antioxidant defenses.

Score	Name	Description
0.641898	Silydianin(positive)	Silydianin is a flavonolignan that acts as a potent antioxidant by scavenging free radicals and enhancing cellular defense mechanisms, while also exhibiting anti-inflammatory and hepatoprotective effects through the modulation of NF-κB and other signaling pathways.
0.640327	Alisol B	Alisol B is a natural triterpenoid that acts as a multi-target agent, primarily functioning as an inhibitor of NF-κ B and TLR4/MyD88 signaling pathways to exert anti-inflammatory effects, while also demonstrating antioxidant activity by scavenging free radicals and enhancing cellular defense mechanisms.
0.640196	simvastatin(positive)	Simvastatin is a competitive inhibitor of HMG-CoA reductase, the rate-limiting enzyme in cholesterol biosynthesis, thereby reducing LDL cholesterol levels and exerting pleiotropic effects such as anti-inflammatory and antioxidant properties through modulation of downstream signaling pathways like Rho/ROCK and NF-κB.
0.637646	Asiatic acid(positive)	Asiatic acid is a natural triterpenoid compound that functions as a multi-target inhibitor and antioxidant, primarily by suppressing NF-κB and MAPK pathways to exert anti-inflammatory effects, activating antioxidant enzymes (e.g., SOD, GPx) to combat oxidative stress, and modulating the PI3K/AKT/mTOR pathway to induce cancer cell apoptosis.

Table S44. TOP50 candidates screened by ElixirSeeker.

(End)
